# Evolutionary history of the Cameroon radiation of puddle frogs (Phrynobatrachidae: *Phrynobatrachus*), with descriptions of two critically endangered new species from the northern Cameroon Volcanic Line

**DOI:** 10.7717/peerj.8393

**Published:** 2020-03-03

**Authors:** Václav Gvoždík, Tadeáš Nečas, Matej Dolinay, Breda M. Zimkus, Andreas Schmitz, Eric B. Fokam

**Affiliations:** 1 Institute of Vertebrate Biology of the Czech Academy of Sciences, Brno, Czech Republic; 2 Department of Zoology, National Museum, Prague, Czech Republic; 3 Department of Botany and Zoology, Masaryk University, Brno, Czech Republic; 4 Museum of Comparative Zoology, Harvard University, Cambridge, MA, USA; 5 Natural History Museum, Geneva, Switzerland; 6 Department of Zoology and Animal Physiology, University of Buea, Buea, Cameroon

**Keywords:** Adamawa Plateau, Amphibians, Bioacoustics, Biodiversity, Biogeography, Diversification, Integrative taxonomy, Rainforest, Species groups, Tadpole description

## Abstract

The Cameroon Volcanic Line, a mountain chain located between West and Central Africa, is a region of numerous endemic diversifications, including of puddle frogs (*Phrynobatrachus*). This study reviews the phylogeny and taxonomy of puddle frogs of the “Cameroon radiation,” which is a clade containing mainly montane but also at least three lowland species. Molecular data revealed a novel evolutionary lineage from high altitudes in the northern part of the mountains. Puddle frogs from the new, minute-sized (SVL < 20 mm) lineage are identified using molecular, morphological and acoustic data and described as two new species, *Phrynobatrachus arcanus* sp. nov. (Gotel Mountains, Cameroon–Nigeria) and *P. mbabo* sp. nov. (Tchabal Mbabo, Cameroon). The tadpole of the first species is also described. Phylogenetic analyses placed the new lineage to the proximity of the recently described lowland small-sized taxa (*P. horsti*, *P. ruthbeateae*). Based on the inferred phylogeny, we propose five species groups within the Cameroon radiation: *P. arcanus*, *P. chukuchuku*, *P. ruthbeateae*, *P. steindachneri*, and *P. werneri*. The taxonomically enigmatic *P. hylaios* is proposed to be a member of the *P. ruthbeateae* species group. The basal radiation evolved during the late Miocene with subsequent diversifications occurring during the Pliocene, while closely related terminal taxa originated during the Pleistocene. We recommend that the newly described species are categorized as Critically Endangered due to their limited ranges and because recent surveys did not identify any individuals at the type localities. This further supports the need for conservation interventions in the mountains of Cameroon and Nigeria.

## Introduction

The Cameroon Volcanic Line (CVL) is situated between biogeographic regions of West and Central Africa and represents the highest mountain chain in this part of Africa (Burke, 2001). The CVL extends along the border area of eastern Nigeria and western Cameroon and is formed by massifs of tectonic and volcanic origin, with the oldest outlier massifs in the northern Cameroon near Lake Chad dated to the Late Cretaceous (Mbowou et al., 2012). The majority of this region was uplifted during the Cenozoic (Oligocene to the Plio-Pleistocene; ca. 30–1 million years ago (Mya)) with a trend of decreasing age of volcanic activity in the southwestern direction (Marzoli et al., 2000). The southern continental part of the CVL is referred to as the Cameroon Highlands and is known for its high level of biodiversity richness and endemism (White, 1983; Stuart, 1986). The Cameroon Highlands are recognized as an ecoregion within the Afromontane forest sub-biome (Burgess et al., 2004). This highland region extends from the Rumpi Hills, the Bakossi Mountains, Mount Kupe and Mount Manengouba in the south of Cameroon into the Obudu Plateau of Nigeria in the north-west. This highland region continues north-east along the Bamenda Highlands (sometimes referred to as the Bamenda-Banso Highlands), Mambilla Plateau in Nigeria to the Gotel Mountains, Tchabal Mbabo, Tchabal Gangdaba and two small outliers further east in the Adamawa Plateau of Cameroon (Stuart, 1986).

The Cameroon Highlands ecoregion is characterized by forest-grassland mosaics above 900 m elevation, with vegetation consisting of submontane forests and grasslands between 900 and 1,800 m, and montane forests and grasslands above 1,800 m (White, 1983). At about 800–1,000 m the vegetation grades into the lowland coastal or more inland Congolian forest ecozones, or the Guineo–Congolian forest-savanna mosaic (Burgess et al., 2004). The natural habitats, mainly montane forests of the Cameroon Highlands, are highly threatened and are being lost due to intensive human activities, especially in the southern portion of the highlands (Cronin et al., 2014; Doherty-Bone & Gvoždík, 2017; Tchassem et al., 2019). The northern parts of the Cameroon Highlands (Mambilla Plateau, Gotel Mountains, Tchabal Mbabo, Tchabal Gangdaba, and Tchabal Nganha) are less densely populated and have always received less biological exploration and conservation attention (Cronin et al., 2014), probably due to their relatively remote locations with more difficult access.

Puddle frogs of the genus *Phrynobatrachus* Günther, 1862 currently contain 92 species that are distributed in various habitats of Sub-Saharan Africa (Frost, 2019; Zimkus, Rödel & Hillers, 2010). Representatives of the genus present in Cameroon are mostly forest-dwelling species phylogenetically belonging to three divergent clades (Zimkus, Rödel & Hillers, 2010). Species confined to the Cameroon Highlands ecozone form a monophyletic group, which contains both montane and lowland taxa (Zimkus, Rödel & Hillers, 2010). Hereafter, we refer to this group as the “*Cameroon radiation*” (CVL clade in Zimkus & Gvoždík (2013)). *Phrynobatrachus batesii* (Boulenger, 1906) is one of the lowland species of the Cameroon radiation distributed in coastal forests from south-eastern Nigeria to northern Gabon. Recently, two more lowland species that are morphologically similar to each other were described (Rödel et al., 2012, 2015) from northwestern Congolian and Atlantic coastal forests in southern Cameroon (*P. ruthbeateae* Rödel, Doherty-Bone, Kouete, Janzen, Garrett, Browne, Gonwouo, Barej, Sandberger, 2012) and the southwestern Republic of the Congo (*P. horsti* Rödel, Burger, Zassi-Boulou, Emmrich, Penner, Barej, 2015). Several montane species from the Cameroon radiation were also recently described, although their phylogenetic positions are not well understood. This is especially true for *P. chukuchuku* Zimkus, 2009, a small-sized species (<20 mm snout-vent length (SVL)) from high elevation wetlands in grassland habitats of Mt. Oku in the Bamenda Highlands (2,160–2,800 m a.s.l.; Doherty-Bone & Gvoždík, 2017). This species morphologically resembles representatives of the *P. werneri* group (see below for details), and was phylogenetically placed with low support either in the sister position to the clade containing the *P. werneri* group + *P. batesii* (Zimkus, 2009; Zimkus, Rödel & Hillers, 2010; Blackburn & Rödel, 2011), in the sister position to the clade containing the *P. steindachneri* complex + *P. cricogaster* (Blackburn, 2010), or in the sister position to all other remaining analyzed species of the Cameroon radiation (Zimkus & Gvoždík, 2013).

A number of species comprise the additional lineages within the Cameroon radiation. We consider the *P. werneri* group to include the following (sub)montane taxa: *P. werneri* (Nieden, 1910) known from relatively scarce findings from a wide range along the Cameroon Highlands; *P. manengoubensis* (Angel, 1940)—supposedly an endemic of Mt. Manengouba, but discussed to be possibly a synonym of *P. werneri* (Amiet, 1975; Grandison, 1985; Amiet, 2004; Zimkus, 2009; Zimkus, Rödel & Hillers, 2010); *P. danko* Blackburn, 2010 known only from the type locality at the western edge of the Mambilla Plateau in Nigeria (Blackburn, 2010); and *P. schioetzi* Blackburn & Rödel, 2011 (previously analyzed under the name *P. werneri* B; Zimkus, Rödel & Hillers, 2010; Zimkus et al., 2012) described from the Nigerian Obudu Plateau, but today known also from the Bamenda Highlands in Cameroon (Doherty-Bone & Gvoždík, 2017). Predominantly submontane *P. cricogaster* Perret, 1957 (Gartshore, 1986) with conspicuous markings on ventrum was found in the sister position to the predominantly montane *P. steindachneri* complex in all previous phylogenetic analyses (Zimkus, 2009; Blackburn, 2010; Zimkus, Rödel & Hillers, 2010; Blackburn & Rödel, 2011; Zimkus et al., 2012; Zimkus & Gvoždík, 2013). The *P. steindachneri* complex is composed by recently described *P. jimzimkusi* Zimkus, Gvoždík & Gonwouo, 2013 from the southern and central parts of the Cameroon Highlands (Mt. Manengouba to Obudu Plateau and Bamenda Highlands), *P. njiomock* Zimkus & Gvoždík, 2013 from and surroundings of Lake Oku on Mt. Oku (Bamenda Highlands), and four evolutionary lineages currently treated under the name *P. steindachneri* Nieden, 1910 known from the central and northern parts of the Cameroon Highlands (Mt. Mbam, Bamenda Highlands, Mambilla Plateau, Gotel Mts., Tchabal Mbabo; Zimkus & Gvoždík, 2013). In addition, based on our investigation of the holotype of *P. hylaios* Perret, 1959, a lowland species described from the borderline between the Congo Basin rainforests and Atlantic coastal forests, and a fact that the taxon was originally described as a subspecies of *P. werneri*, we suppose that this species probably also represents a member of the Cameroon radiation.

In 2000, 2009, and 2016, we surveyed amphibians in rarely visited and relatively remote northern regions of the Cameroon Highlands, including montane forests and grasslands of the Tchabal Mbabo massif on the Adamawa Plateau of Cameroon and Gotel Mts. in the border region of Cameroon and Nigeria. During our fieldwork, we collected a series of a small-sized *Phrynobatrachus*, which morphologically resembled *P. chukuchuku* or members of the *P. werneri* group but did not correspond to any known species. The aim of this study was to put these specimens into a phylogenetic framework with all available taxa of the Cameroon radiation, both montane and lowland, and to compare them morphologically with all montane taxa to gain new insights into the evolutionary history and diversification of this western Central African clade of *Phrynobatrachus*.

## Materials and Methods

### Taxon sampling and molecular markers

We investigated nine specimens of the putative new species in a phylogenetic context, including specimens from the Gotel Mts. (3 males, 1 female, 1 juvenile metamorph, 1 tadpole) and Tchabal Mbabo (3 females; sequence data from male specimens did not amplify). We included all available DNA sequence data from GenBank of the taxa known to form the Cameroon radiation (Zimkus & Gvoždík, 2013), which contained montane taxa plus lowland *P. batesii* and recently described lowland *P. horsti* (Rödel et al., 2015). The latter was phylogenetically analyzed under the name “*P. ruthbeateae*” by Zimkus & Gvoždík (2013) before the species was formally described, and under this name was shown to be a part of the Cameroon radiation. The “true” *P. ruthbeateae* has never been analyzed in a phylogenetic framework; therefore, we also included genetic data of this species, including from the holotype, which was available in GenBank (Rödel et al., 2012). As additional material, we also included several new samples of the taxa from the Cameroon radiation: *P. batesii* (*n* = 1), *P. cricogaster* (*n* = 3), *P. horsti* (*n* = 5; including paratype, ZMB 81699), *P. manengoubensis* (*n* = 4), and *P. schioetzi* (*n* = 1), for which the *16S* barcode was sequenced (see below). Outgroups were selected following the published phylogeny reconstruction of *Phrynobatrachus*: *P. africanus* (Central Africa) being the sister taxon, and *P. tokba* (West Africa) and *P. auritus* (Central Africa) representing more distant taxa from within a common clade (Zimkus, Rödel & Hillers, 2010; Zimkus et al., 2012). Details on the new species material and genetically examined additional specimens are given in Table 1. Sequence data downloaded from GenBank are shown in phylogenetic trees with their GenBank accession numbers. The examined material was preserved in 75% ethanol (post-metamorph vouchers), 8% formalin (tadpole vouchers) or 96% ethanol (tissues) and deposited in the collections of the National Museum in Prague (NMP); “Zoologisches Forschungsmuseum Alexander Koenig”, Bonn (ZFMK); “Museum für Naturkunde Berlin” (ZMB); and the “Institut National de Recherche en Sciences Exactes et Naturelles”, Brazzaville (IRSEN). For the IRSEN material, the MBUR numbers refer to the field numbers as no formal accession numbers are available. The numbers starting with “vg” refer to the tissue material (no vouchers available) from the Václav Gvoždík’s collection.

**Table 1:** Material examined of the two new species and genetically examined additional material. Specimen catalogue numbers, collecting localities with geographic coordinates, and GenBank accession numbers, holotypes in bold. See “Material and Methods” for catalogue abbreviations, the standard ones follow Sabaj (2019).

Species	Catalogue No.	Locality	GPS (°N)	GPS (°E)	Elevation	Sex/Stage	Type	Morphology	*12–16S*	*RAG1*
***P. arcanus* sp. nov.**	**NMP-P6V 74603/1**	**Gotel Mts., Mt. Gangirwal, site 2 (Cameroon/Nigeria)**	**7.0398**	**11.7068**	**2250 m**	**male**	**holotype**	**external + μCT**	**MN158662**	**MN158684**
*P. arcanus* sp. nov.	NMP-P6V 74603/2	Gotel Mts., Mt. Gangirwal, site 2 (Cameroon/Nigeria)	7.0398	11.7068	2,250 m	female	paratype	external + μCT	MN158663	MN158685
*P. arcanus* sp. nov.	NMP-P6V 74603/3	Gotel Mts., Mt. Gangirwal, site 2 (Cameroon/Nigeria)	7.0398	11.7068	2,250 m	male	paratype	external	MN158664	MN158686
*P. arcanus* sp. nov.	NMP-P6V 74603/4	Gotel Mts., Mt. Gangirwal, site 2 (Cameroon/Nigeria)	7.0398	11.7068	2,250 m	male	paratype	external	–	–
*P. arcanus* sp. nov.	NMP-P6V 74603/5	Gotel Mts., Mt. Gangirwal, site 2 (Cameroon/Nigeria)	7.0398	11.7068	2,250 m	male	paratype	external	–	–
*P. arcanus* sp. nov.	NMP-P6V 74603/6	Gotel Mts., Mt. Gangirwal, site 2 (Cameroon/Nigeria)	7.0398	11.7068	2,250 m	male	paratype	external	–	–
*P. arcanus* sp. nov.	NMP-P6V 74603/7	Gotel Mts., Mt. Gangirwal, site 2 (Cameroon/Nigeria)	7.0398	11.7068	2,250 m	juvenile	–	external	–	–
*P. arcanus* sp. nov.	NMP-P6V 74603/8	Gotel Mts., Mt. Gangirwal, site 2 (Cameroon/Nigeria)	7.0398	11.7068	2,250 m	juvenile	–	external	MN158665	MN158687
*P. arcanus* sp. nov.	NMP-P6V 74604	Gotel Mts., Mt. Gangirwal, site 1 (Cameroon/Nigeria)	7.0307	11.7021	1940 m	male	paratype	external	MN158666	MN158688
*P. arcanus* sp. nov.	vg09-173	Gotel Mts., Mt. Gangirwal, site 2 (Cameroon/Nigeria)	7.0398	11.7068	2,250 m	tadpole	–	–	MN158667	MN158689
*P. arcanus* sp. nov.	NMP-P6V 75866/A	Gotel Mts., Mt. Gangirwal, site 2 (Cameroon/Nigeria)	7.0398	11.7068	2,250 m	tadpole	–	external	–	–
*P. arcanus* sp. nov.	NMP-P6V 75866/B	Gotel Mts., Mt. Gangirwal, site 2 (Cameroon/Nigeria)	7.0398	11.7068	2,250 m	tadpole	–	external	–	–
*P. arcanus* sp. nov.	NMP-P6V 75866/C	Gotel Mts., Mt. Gangirwal, site 2 (Cameroon/Nigeria)	7.0398	11.7068	2,250 m	tadpole	–	external	–	–
*P. arcanus* sp. nov.	ZFMK 47955	Gotel Mts., Mt. Gangirwal (Nigeria)	7.03	11.70	~2,300 m	female	–	external	–	–
*P. arcanus* sp. nov.	ZFMK 47956	Gotel Mts., Mt. Gangirwal (Nigeria)	7.03	11.70	~2,300 m	female	paratype	external	–	–
*P. arcanus* sp. nov.	ZFMK 47957	Gotel Mts., Mt. Gangirwal (Nigeria)	7.03	11.70	~2,300 m	female	–	external	–	–
*P. arcanus* sp. nov.	ZFMK 47958	Gotel Mts., Mt. Gangirwal (Nigeria)	7.03	11.70	~2,300 m	male	paratype	external	–	–
***P. mbabo* sp. nov.**	**ZFMK 75726**	**Tchabal Mbabo, 5 km NEE of Foungoy (Cameroon)**	**7.2518**	**12.0597**	**2,060 m**	**female**	**holotype**	**external + μCT**	**MN158668**	**MN158690**
*P. mbabo* sp. nov.	ZFMK 75676	Tchabal Mbabo, 5 km NEE of Foungoy (Cameroon)	7.2518	12.0597	2,060 m	male	paratype	external + μCT	–	–
*P. mbabo* sp. nov.	ZFMK 75686	Tchabal Mbabo, 5 km NEE of Foungoy (Cameroon)	7.2518	12.0597	2,060 m	female	paratype	external	MN158669*	–
*P. mbabo* sp. nov.	ZFMK 75650	Tchabal Mbabo, 5 km NEE of Foungoy (Cameroon)	7.2518	12.0597	2,060 m	female	–	external	MN158670*	–
*P. mbabo* sp. nov.	ZFMK 75677	Tchabal Mbabo, 5 km NEE of Foungoy (Cameroon)	7.2518	12.0597	2,060 m	female	paratype	external	–	–
*P. mbabo* sp. nov.	ZFMK 75683	Tchabal Mbabo, 5 km NEE of Foungoy (Cameroon)	7.2518	12.0597	2,060 m	male	–	external	–	–
*P. mbabo* sp. nov.	ZFMK 75728	Tchabal Mbabo, 5 km NEE of Foungoy (Cameroon)	7.2518	12.0597	2,060 m	female	paratype	external	–	–
*P. mbabo* sp. nov.	ZFMK 75729	Tchabal Mbabo, 5 km NEE of Foungoy (Cameroon)	7.2518	12.0597	2,060 m	female	paratype	external	–	–
*P. batesii*	NMP-P6V 74620	Bakossi Mts., Edib–Messaka (Cameroon)	4.9642	9.6475	1,170 m	male	–	external	MN158671*	–
*P. cricogaster*	NMP-P6V 73393/1	Bamenda Highlands, Mejung, Bamo Forest (Cameroon)	6.1326	10.1781	890 m	male	–	external	MN158672*	–
*P. cricogaster*	NMP-P6V 73393/5	Bamenda Highlands, Mejung, Bamo Forest (Cameroon)	6.1326	10.1781	890 m	male	–	external	MN158673*	–
*P. cricogaster*	NMP-P6V 74642	Bakossi Mts., near Lake Edib, site 2 (Cameroon)	4.9599	9.6572	1,280 m	male	–	external	MN158674*	–
*P. horsti*	ZMB 81699	Sintoukola, Mayombe, Yombo River (Congo Rep.)	−4.3906	11.6739	30 m	male	paratype	–	MN158675*	MN158691
*P. horsti*	IRSEN-MBUR 3028	Sintoukola, Mayombe, Yombo River (Congo Rep.)	−4.3906	11.6739	30 m	?	–	–	KR827543*	MN158692
*P. horsti*	IRSEN-MBUR 3029	Sintoukola, Mayombe, Yombo River (Congo Rep.)	−4.3906	11.6739	30 m	?	–	–	MN158676*	MN158693
*P. horsti*	IRSEN-MBUR 3030	Sintoukola, Mayombe, Yombo River (Congo Rep.)	−4.3906	11.6739	30 m	?	–	–	MN158677*	MN158694
*P. horsti*	IRSEN-MBUR 3031	Sintoukola, Mayombe, Yombo River (Congo Rep.)	−4.3906	11.6739	30 m	?	–	–	MN158678*	MN158695
*P. manengoubensis*	NMP-P6V 74607	Bakossi Mts., Lake Edib, shore (Cameroon)	4.9594	9.6533	1,260 m	female	–	external	MN158679*	–
*P. manengoubensis*	NMP-P6V 74644	Bakossi Mts., near Lake Edib, site 1 (Cameroon)	4.9605	9.6524	1,290 m	female	–	external	MN158680*	–
*P. manengoubensis*	vg09-243	Bakossi Mts., Lake Edib, shore (Cameroon)	4.9594	9.6533	1,260 m	juvenile	–	–	MN158681*	–
*P. manengoubensis*	vg09-244	Bakossi Mts., Lake Edib, shore (Cameroon)	4.9594	9.6533	1,260 m	juvenile	–	–	MN158682*	–
*P. schioetzi*	NMP-P6V 73438	Bamenda Highlands, Kedjom Keku (Cameroon)	6.0906	10.3025	2,190 m	female	–	external	MN158683*	–

**Note:**

* *16S* only.

We generated three datasets, two consisting of mitochondrial DNA (mtDNA) fragments and one from the nuclear recombination-activating gene (*RAG1*). One mitochondrial dataset was composed of a matrix of all available sequences comprising fragments within a mitochondrial DNA portion spanning 12S–16S rRNA (*12S–16S*), hereafter as the “*individuals-mt-dataset*”. As the individuals-mt-dataset contained a high level of missing data, we prepared also a dataset containing all sequences of our putative new taxa from the Gotel Mts. and Tchabal Mbabo complemented by representatives of all taxa with sequence data investigated thus far, including four lineages of *P. steindachneri* (Zimkus & Gvoždík, 2013) from four massifs inclusive of the syntopic sites with the putative new species in the Gotel Mts. and Tchabal Mbabo (hereafter the four lineages as *P. steindachneri* A, B, C, D). *Phrynobatrachus hylaios* was not available as all sequences of “*P*. cf. *hylaios*” (or “*P*. cf. *hylaois*” sic) deposited in GenBank are of a taxon belonging to the clade containing *P. latifrons* Ahl, 1924 (Zimkus, Rödel & Hillers, 2010), and does not morphologically conform to the holotype of *P. hylaios* (more details in Discussion). Longer sequences (spanning the *12S–16S* region) and type material were preferred, genetic data of holotypes of the following taxa were included: *P. danko*, *P. jimzimkusi*, *P. njiomock*, and *P. ruthbeateae*. Hereafter, this dataset is referred to as the “*species-mt-dataset*”. The third, nuclear RAG1 dataset consisted of *RAG1* sequences of all available *Phrynobatrachus* taxa from GenBank (majority from Zimkus, Rödel & Hillers (2010)) supplemented by new sequences of our putative new taxa and recently described *P. horsti* (*RAG1* not yet analyzed). *RAG1* sequences of the taxa of the Cameroon radiation were phased into gametic haplotypes (see below).

### Laboratory procedures, DNA alignment and phasing

Total genomic DNA was extracted from tissue samples using commercial kits following the manufacturers’ protocols. In mtDNA, we targeted a portion of an approximately 2 kb-long *12S–16S* fragment. The whole fragment was amplified in two overlapping pieces using primers 12Sa (5′-CTGGGATTAGATACCCCACTA-3′; adapted from Kocher et al. (1989)) and 16SH (or H3296: 5′-GCTAGACCATKATGCAAAAGGTA-3′; Richards & Moore, 1996), and the newly designed primer 12SbLin (5′-ACCGCCCGTCACCCTCTT-3′) and 16SH1 (5′-CCGGTCTGAACTCAGATCACGT-3′; Palumbi et al., 1991). We were not able to obtain good quality sequences for the entire *12S–16S* fragment for some specimens. For the most problematic specimens (e.g., older museum samples) and the additional material, we aimed to obtain a fragment (ca. 510 bp when trimmed) of 16S rRNA (*16S*) widely used in the amphibian DNA barcoding (Vences et al., 2012; primers 16SL1 and 16SH1; 16SL1: 5′-CGCCTGTTTAACAAAAACAT-3′, adapted from Palumbi et al., 1991). PCR amplification involved an initial cycle of denaturation at 94 °C for 10–15 min, and 35 subsequent cycles of 94 °C for 30–60 s, 55 °C for 30 s and 72 °C for 60–90 s, followed by a final extension step of 72 °C for 10 min. The nuclear *RAG1* was amplified by the primers and following the protocol of Zimkus, Rödel & Hillers (2010). Sequencing was carried out using the PCR primers by Macrogen Europe B.V. (https://dna.macrogen-europe.com). Sequences have been deposited in GenBank: MN158662–MN158695.

Mitochondrial DNA sequences of the individuals-mt-dataset were aligned using MAFFT v7 (Katoh & Standley, 2013) with default setting as incorporated in Geneious v8.1 (Biomatters, available from www.geneious.com) and checked by eye, having 2,459 aligned sites. In the species-mt-dataset, ambiguously aligned positions were eliminated by Gblocks v. 0.91b under options for a less stringent selection (Castresana, 2000), producing a final alignment of 1,806 bp. *RAG1* was aligned manually as it contained no gaps and produced an alignment of 921 bp. Gametic haplotypes of the ingroup (Cameroon radiation) were inferred by the coalescent-based Bayesian algorithm of Phase 2.1 (Stephens, Smith & Donnelly, 2001; Stephens & Scheet, 2005) as implemented in DnaSP 5.10 (Librado & Rozas, 2009). The analysis was repeated three times with different seeds for the random-number generator to check if the phase estimates are consistent across the runs according to goodness-of-fit values. Each run was conducted under the parent-independent mutation model with a burn-in-period of 100 iterations, which was followed by 1,000 iterations. Results with a probability >0.70 were accepted. No stop codons were detected in the haplotypes of *RAG1* as checked by translation with the standard genetic code. The phased *RAG1* ingroup sequences were combined with all other *Phrynobatrachus RAG1* sequences available in GenBank (unphased) together with *Ptychadena* (Ptychadenidae; GenBank GU457783) and *Cardioglossa* (Arthroleptidae; GenBank GU457592) outgroups to find out the position of the Cameroon radiation within the *Phrynobatrachus* genus.

### Gene trees, species tree and divergence dating

Gene trees were inferred by the maximum-likelihood (ML) and Bayesian inference (BI) using PhyML 3 (Guindon et al., 2010) and MrBayes 3.2 (Ronquist et al., 2012). The best-fit nucleotide substitution models were selected by the Smart Model Selection (SMS v1.8) in PhyML (Lefort, Longueville & Gascuel, 2017), and the GTR+G+I model was selected for all datasets except of the individuals-mt-dataset where the TrN+G+I model was applied. For the MrBayes analysis of *RAG1*, the best-fit model was selected together with the best-fit partition for codon positions in PartitionFinder v2 (Lanfear et al., 2017). The model HKY+G+I and HKY+G was applied for the 1st+2nd codon position and 3rd codon position, respectively. All MrBayes analyses were performed with two runs of four Markov chains, which were run for 6 million generations and samples saved every 100th generation. Plots of log-likelihood scores were examined in Tracer 1.7 (Rambaut et al., 2018; all parameters had effective sample size (ESS) > 200) to verify that the analyses reached stationarity, and the chain convergence was confirmed by the convergence diagnostics. The first 25% of the sampled trees were discarded as a burn-in and a 50% majority-rule consensus tree was produced from the remaining trees. In PhyML, the best option of a combination of the nearest neighbor interchange and the subtree pruning and regrafting algorithm of tree improvement and optimization of the topology and branch lengths were applied. The nodal support was assessed by 100 bootstrap pseudoreplicates. Clades supported with ML bootstrap values *P* ≥ 70 and BI posterior probability (pp) values *P* ≥ 0.95 were considered highly supported (Huelsenbeck & Rannala, 2004). Mean genetic uncorrected *p*-distances based on *16S* were calculated among and within taxa in MEGA X (Kumar et al., 2018).

In addition to gene trees, a dated coalescent-based species tree was inferred in *BEAST 1.8 (Heled & Drummond, 2010; Drummond et al., 2012) based on mtDNA (*12S–16S*) and phased *RAG1*. Because only taxa with all loci available may be analyzed in *BEAST, *P. danko* and *P. ruthbeateae* were not included due to missing data for *RAG1*. *Phrynobatrachus steindachneri* was also not included as the only specimen available for *RAG1* (MCZ A-138104; Mt. Oku) might represent a hybrid as shown by the analysis of phased *RAG1* haplotypes (see below). *Phrynobatrachus africanus* (MCZ A-136944) was used as outgroup. Based on preliminary analyses, a strict molecular clock was rejected using the coefficient of variation for the clock rate (values above 0.1; Drummond et al., 2006; Drummond & Bouckaert, 2015), therefore an uncorrelated lognormal relaxed clock was used. Since no fossils were available to serve as calibration points, we applied an estimate of the substitution rate for the mtDNA fragment as used in another African natatanuran genus, *Ptychadena* (Zimkus et al., 2017), using a normal distribution prior with a mean of 0.0125 substitutions/site/My and standard deviation of 0.001. The clock rate for *RAG1* was estimated from mtDNA using the 1/x prior distribution. The Yule species-tree prior, the ploidy of *12S–16S* to mitochondrial (haploid) and *RAG1* to autosomal nuclear (diploid) were set. To avoid overparametrization codon-position partitions (*RAG1*) were not applied, and the substitution models HKY+I and TrN+G+I (as selected by jModelTest 2 (Darriba et al., 2012) for the *BEAST datasets) were used for *RAG1* and *12S–16S*, respectively. The final analysis was run in triplicate to check for consistency among runs, each run with 40–150 million generations and sampled in a frequency to save 10,000 samples. Convergence, stationarity and the appropriate number of steps to be discarded as burn-in (10%) were assessed using Tracer 1.7 (Rambaut et al., 2018), and the posterior distribution of trees was evaluated with DensiTree 2.2 (Bouckaert & Heled, 2014). Satisfactorily high ESS values for all parameters were obtained. The post burn-in samples of the three runs were combined in the BEAST module LogCombiner. The output of 27,000 sampled trees was uploaded to another BEAST module, TreeAnnotator, to infer the final species tree as a maximum clade credibility tree (MCCT) with node ages represented by median heights and confidence intervals with 95% highest posterior densities (HPD).

### Morphology

#### External morphology of adults

Males were identified by secondary sexual characteristics (e.g., presence of vocal sac/folds in the gular region, nuptial pad) or presence of testes, when dissected. Females were identified by smooth gular region in specimens larger than mature males or presence of eggs or ovaries, when dissected. The following measurements were taken with a digital caliper or dissecting microscope in each adult specimen to the nearest 0.1 mm in the standardized manner: snout-vent length (SVL), from snout tip to posterior margin of vent; snout-urostyle length (SUL), from snout tip to posterior margin of urostyle; head width (HW), at greatest head width in close proximity to angle of jaws; head length (HDL), from snout tip to angle of jaw; tympanum diameter (TD), at greatest tympanum width; eye diameter (ED), at greatest anterior-posterior diameter of upper eyelid; interorbital distance (IOD), at shortest distance between upper eyelids; eye anterior distance (EAD), between anterior corners of eyes; eye posterior distance (EPD), between posterior corners of eyes; internarial distance (IND), between midpoints of nostrils; snout length (SL), from anterior corner of eye to snout tip; snout-nostril distance (SNL), from midpoint of external naris to snout tip; eye-nostril distance (ENL), from anterior border of eye to midpoint of external naris; humerus length (HL), from body wall to outer edge of elbow; radioulna length (RL), from elbow to proximal edge of most proximal palmar tubercule; manual digit 1–4 length (MD1–4) and pedal digit 1–5 length (PD1–5), from proximal edge of most proximal subarticular tubercule to distal tip; femur length (FL), from center of vent to outer edge of knee; tibiofibula length (TL), from knee to outer edge of tibiotarsal articulation; tarsus and foot length (FTL), from outer edge of tibiotarsal articulation to distal tip of fourth (= longest) pedal digit; inner metatarsal tubercule length (IMTL) and outer metatarsal tubercule length (OMTL), parallel to hind limb axis at greatest lengths.

### Osteology—cranial morphology

The micro computed tomography (μCT) approach was applied to evaluate cranial morphology of adults—holotypes (NMP-P6V 74603/1, ZFMK 75726) and allotypes (paratypes of the opposite sex to holotypes; NMP-P6V 74603/2, ZFMK 75676). Specimens were treated with phosphotungstic acid following the protocol developed by Metscher (2009) and scanned with a microCT SkyScan 1172 (Bruker) at the Department of Paleontology, National Museum in Prague. Images were reconstructed in N-Recon (Bruker), and virtual sections were analyzed in Avizo 9.1 (Thermo Fisher Scientific, Waltham, MA, USA). The osteological terminology follows Drewes (1984) and Duellman & Trueb (1986). Raw μCT data has been deposited in MorphoSource: S27195, S27196, S27198, S27200 (www.morphosource.org).

### External morphology of tadpoles

Four larvae/tadpoles were collected at the type locality of the new species from the Gotel Mts. Species identification was confirmed based on DNA sequencing of one tadpole (vg09-173) stored in 96% ethanol. The remaining three formalin-fixed tadpoles (NMP-P6V 75866/A–C) were examined morphologically and measured in the standardized manner under a dissecting microscope (or the total length using a digital caliper) to the nearest 0.1 mm: total length (Ltot), from snout tip to tip of tail; body length (BL), from snout tip to base of tail; tail length (TAL), from base of tail to its tip; body height (BH), at point of spiraculum; body width (BW), at widest point of body; tail width (TW), at tail base; (tail height at tail base (THB), at tail base; dorsal fin height (DFH), at highest point of dorsal fin; ventral fin height (VFH), at highest point of ventral fin; maximum height of tail (THM), at highest point of tail and fins; eye diameter (ED), at greatest anterior-posterior diameter; eye distance (ECD), between centers of eyes; snout-eye distance (SED), from snout tip to midpoint of eye; oral disc width (ODW), widest transversal diameter; spiracle length (SL), from base to tip; snout-spiracle distance (SSD), from snout tip to spiracle base. The larval morphological terminology follows Altig & McDiarmid (1999) and Altig (2007).

### Bioacoustics

Advertisement calls of the new species from the Gotel Mts. were recorded in the field using a digital recorder (Edirol R-09HR) and an external electret condenser microphone (Sony ECM-MS907). Ambient temperature was measured in the exact place occupied by the calling male using a digital thermometer (Viking AB 06912). Recordings were made at the type locality (Mount Gangirwal, pond in small stream surrounded by shrubs on a grassland slope, 7.0398°N, 11.7068°E, 2,250 m), on 5 November 2009 at approximately 16:00. The advertisement call of the new species from Tchabal Mbabo was recorded and documented by Amiet & Goutte (2017; as *Phrynobatrachus* sp. 5). The call recordings (made at the frequency 44.1 kHz, 16-bit sampling rate in the Gotel Mts.) were analyzed using Raven Lite 2.0 (Center for Conservation Bioacoustics, 2016) and SoundRuler 0.9.6 (Gridi-Papp, 2007), with spectrograms at the Hanning window function and 512 bands resolution. Oscillograms and spectrograms of the advertisement call of a single male were inspected and the following parameters measured/counted/calculated: note (click) duration (ms), duration of a note group (s), number of notes in a note group, note repetition rate (notes/s), and dominant frequency (kHz). Measurements were taken with precisions 1 ms and 10 Hz, averaged values were taken from all notes within a note group (note groups within a note series). Bioacoustic methodology and terminology follow recommendations by Köhler et al. (2017). The recording has been deposited in FonoZoo with the reference number 11489 (http://www.fonozoo.com/).

### Nomenclatural note

The electronic version of this article in Portable Document Format (PDF) will represent a published work according to the International Commission on Zoological Nomenclature (ICZN), and hence the new names contained in the electronic version are effectively published under that Code from the electronic edition alone. This published work and the nomenclatural acts it contains have been registered in ZooBank, the online registration system for the ICZN. The ZooBank LSIDs (Life Science Identifiers) can be resolved and the associated information viewed through any standard web browser by appending the LSID to the prefix http://zoobank.org/. The LSID for this publication is: urn:lsid:zoobank.org:pub:71CF018D-9E8B-46CD-A6EA-1F9CCB809765. The online version of this work is archived and available from the following digital repositories: PeerJ, PubMed Central and CLOCKSS.

### Collection of specimens

The field research was conducted under the permissions issued by the Cameroon Ministry of Scientific Research and Innovation (MINRESI: No. 00039/MINRESI/B00/C00/C10/C12) and Ministry of Forestry and Wildlife (MINFOF: Nos. 1010/PRBS/MINFOF/SG/DFAP/SDVEF/SC and 1099/PRS/MINFOF/SG/DFAP/SDVEF/SC).

## Results

### Mitochondrial DNA

**Figure 1:**
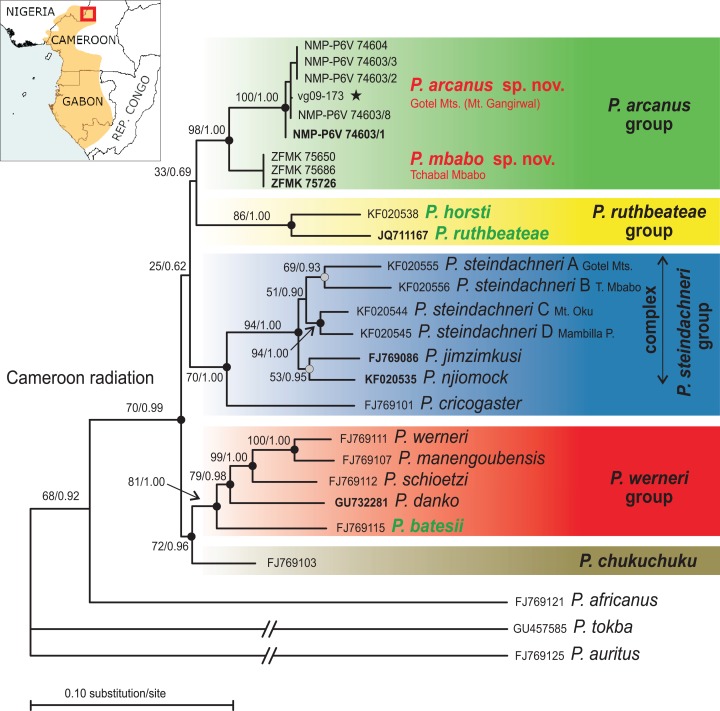
Phylogenetic tree (ML) based on the *species-mt-dataset* (*12S–16S*) and map showing the known distribution range of the Cameroon radiation of *Phrynobatrachus* (orange area). Numbers at nodes are support values (ML bootstrap/Bayesian posterior probabilities) and dots represent relative support values: black dots = high support, gray dots = intermediate support or high only in one method. Background color denotes species groups (note that the *P. steindachneri* group also contains *P. cricogaster*, while the *P. steindachneri* complex only contains the remaining taxa/lineages). Names of lowland species in green color, and the new lineage/species from the Gotel Mountains and Tchabal Mbabo in red color. Specimen codes not starting with NMP, vg, or ZFMK are GenBank accession numbers. Specimen codes in bold are holotypes. Star denotes the tadpole specimen. Red rectangle in the inset map shows the region of the northern Cameroon Volcanic Line.

The mitochondrial DNA phylogenetic tree based on the species-mt-dataset (Fig. 1), where ambiguously aligned positions were removed, supports four major clades: (1) one clade composed of the nine specimens of the putative new species from the Gotel Mts. and Tchabal Mbabo (ML bootstrap/BI pp: 98/1.00), (2) the *P*. *ruthbeateae*–*P. horsti* clade (86/1.00), (3) the *P. cricogaster*–*P*. *steindachneri*-complex clade (70/1.00), and (4) a clade comprising the *P. werneri* group (inclusive *P. batesii* with high support 81/1.00) and *P. chukuchuku* (72/0.96). Mutual relationships among the major clades are not resolved, which is also true for the individuals-mt-dataset containing all positions (Fig. S1). In the latter dataset, the general topology is the same as the species-mt-dataset with the main difference being the position of *P. chukuchuku* as the sister taxon (ML bootstrap support 76) to the clade (84) containing the *P. ruthbeateae*–*P. horsti* clade and the putative new species. The lowland *P. batesii* is again in the sister taxon position to the *P. werneri* group with a high ML bootstrap support (91). The *P. ruthbeateae*–*P. horsti* clade is again highly supported (82) as well as the clade containing the putative new species (99). When inspecting the mtDNA alignment before the removal of ambiguously aligned positions, a unique insertion in *16S* (4–7 bp) in all *P. werneri* group taxa and in *P. batesii* was identified. This insertion is not present in *P. chukuchuku*. The individuals-mt-dataset inferred six lineages within the *P. steindachneri* complex, two corresponding to the recently described taxa, *P. jimzimkusi* and *P. njiomock*, and four to *P. steindachneri* A, B, C, D. Mutual relationships among the six lineages were not completely resolved, with a high support for the sister relationship of *P. steindachneri* C from Mt. Oku and D from Mambilla Plateau (94/1.00 in species-mt-dataset; 89 in individuals-mt-dataset, respectively), high support in BI for the sister relationship of *P. jimzimkusi* and *P. njiomock* (53/0.95; –), and moderate supports for the sister relationship of *P. steindachneri* A from Gotel Mts. and B from Tchabal Mbabo (69/0.93; 40). The four *P. steindachneri* lineages form a clade with a rather low support (51/0.90; 38). The nine specimens of the putative new species form two, moderately divergent clades in both mtDNA datasets, one containing specimens from the Gotel Mts., second from Tchabal Mbabo. Mitochondrial DNA (*16S*) genetic distances among taxa are given in Table 2.

**Table 2:** Genetic distances. Averaged among and within (in bold) taxa based on the fragment of the mitochondrial 16S rRNA gene (uncorrected *p*; expressed as percentages).

	1	2	3	4	5	6	7	8	9	10	11	12	13	14	15	16	17	18	19	20
1. *P. arcanus* sp. nov.	**0.2**																			
2. *P. mbabo* sp. nov.	1.2	**0.0**																		
3. *P. horsti*	3.8	3.1	**0.6**																	
4. *P. ruthbeateae*	4.3	3.7	3.4	**1.8**																
5. *P. chukuchuku*	3.3	2.8	3.8	4.5	**0.0**															
6. *P. steindachneri* A	4.9	4.1	4.8	5.8	5.7	**0.0**														
7. *P. steindachneri* B	5.1	4.8	4.0	5.5	5.6	3.4	**0.2**													
8. *P. steindachneri* C	3.9	3.2	3.9	5.4	4.2	2.6	2.7	**0.1**												
9. *P. steindachneri* D	4.1	3.4	4.4	5.9	4.8	2.7	3.1	1.3	**0.3**											
10. *P. njiomock*	4.2	3.5	4.0	5.0	3.7	3.5	3.0	2.2	2.6	**0.1**										
11. *P. jimzimkusi*	4.2	3.5	4.1	5.2	4.1	3.7	4.0	2.6	3.0	2.1	**0.4**									
12. *P. cricogaster*	4.2	4.4	5.4	5.3	5.2	7.3	5.8	5.9	6.5	5.5	5.7	**0.4**								
13. *P. werneri*	4.8	4.2	4.1	5.1	3.9	6.7	5.8	5.5	5.8	4.8	5.1	5.4	**1.0**							
14. *P. manengoubensis*	5.6	4.9	4.5	5.7	4.5	7.5	6.5	6.2	6.5	5.7	6.1	6.2	1.5	**0.0**						
15. *P. schioetzi*	4.1	3.5	3.9	4.7	3.3	5.7	5.5	4.8	5.0	4.9	4.9	5.2	2.5	3.3	**0.4**					
16. *P. danko*	5.0	4.7	4.9	5.7	4.5	7.0	6.4	6.1	6.5	5.6	6.4	5.7	4.4	4.1	3.9	**0.1**				
17. *P. batesii*	5.1	4.8	4.9	5.1	4.3	7.0	6.4	6.3	6.8	5.6	6.3	6.0	4.2	4.5	3.5	3.9	**1.7**			
Outgroups																				
18. *P. africanus*	7.9	8.1	8.8	8.7	8.7	10.4	9.0	8.7	9.7	8.0	9.2	7.0	9.6	9.8	9.8	9.0	8.8	**N/A**		
19. *P. tokba*	10.6	10.4	10.9	10.5	10.4	11.0	11.3	11.5	11.6	11.4	11.7	10.5	11.5	12.0	10.1	10.2	10.1	11.2	**N/A**	
20. *P. auritus*	12.5	12.6	11.4	11.8	12.8	12.4	11.5	12.9	12.9	12.0	13.3	11.5	12.4	13.0	11.8	12.7	11.5	14.6	13.2	**N/A**

### Nuclear DNA

**Figure 2:**
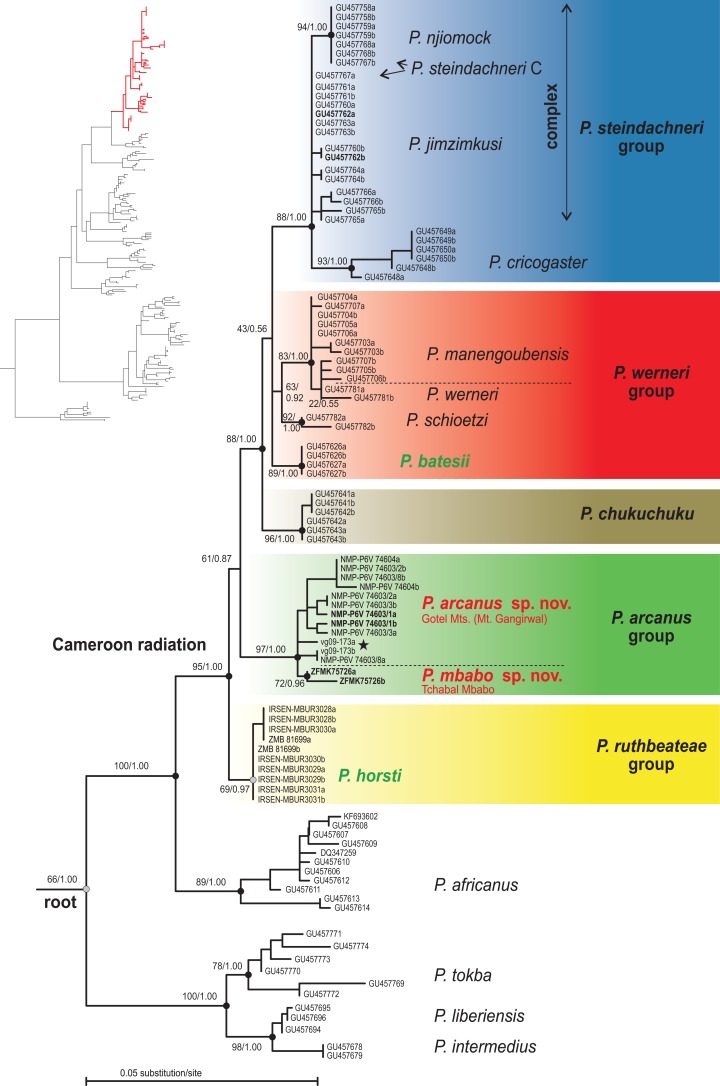
Phylogenetic tree (ML) based on the nuclear *RAG1* dataset of the Cameroon radiation of *Phrynobatrachus* and related lineages. Numbers near nodes are support values (ML bootstrap/Bayesian posterior probabilities), and dots are interpretations of the support: black dots = high support, gray dots = intermediate support or high only in one method. Background color denotes species groups. Names of lowland species in green color, and the new lineage/species from the Gotel Mountains and Tchabal Mbabo in red color. Specimen codes not starting with NMP, vg, ZFMK, IRSEN, or ZMB are GenBank accession numbers. Nucleotide sequences of the taxa of the Cameroon radiation were phased into gametic haplotypes, the two alleles differentiated with ‘a’ and ‘b’. Specimen codes in bold are holotypes. Star denotes the tadpole specimen. Red lineage in the inset phylogenetic tree (genus *Phrynobatrachus*) indicates the Cameroon radiation clade.

The nuclear DNA phylogenetic tree (Fig. 2) supported the sister relationship between the Cameroon radiation and western Central African *P. africanus* (100/1.00), and both clades to West African forest species (*P. tokba*, *P. liberiensis*, *P. intermedius*; 66/1.00). The phasing into gametic haplotypes of the Cameroon radiation ingroup solved 90.9% of the heterozygous sites with a probability >0.70, the remaining sites (*n* = 5) were coded with the IUPAC ambiguity codes. The phased-*RAG1* dataset of the Cameroon radiation inferred seven highly supported clades: *P. horsti* (69/0.97), clade containing the new putative species (97/1.00), *P. chukuchuku* (96/1.00), *P. batesii* (89/1.00), *P. schioetzi* (92/1.00), *P. werneri* + *P. manengoubensis* (83/1.00), and *P. steindachneri* complex + *P. cricogaster* (88/1.00). Five of these seven clades from a major clade (88/1.00) keeping the two remaining, clearly separated clades outside (*P. horsti* and the putative new species). Haplotypes of the *P. werneri* group and *P. batesii* group together form a haplotype cluster. Haplotypes of *P. werneri* and *P. manengoubensis* are paraphyletic in respect to each other. Haplotypes of *P. cricogaster* form a clade (93/1.00), which is embedded within the clade formed by haplotypes of the *P. steindachneri* complex. Within this species complex, only *P. jimzimkusi*, *P. njiomock*, and one individual of *P. steindachneri* C (Mt. Oku) were available in *RAG1* analyses, with the *P. steindachneri* C individual having heterozygous *RAG1* composed of gametic haplotypes “typical” for *P. jimzimkusi* and *P. njiomock*. Thus, the “*P. steindachneri* C” individual (based on mtDNA) might represent a hybrid. The Lake Oku endemic, *P. njiomock*, forms a subclade (94/1.00) within the common clade. Haplotypes of the specimens of the putative new species from Tchabal Mbabo form a clade (72/0.96), which is embedded within the clade formed by haplotypes of the specimens of the putative new species from the Gotel Mts.

### Taxonomy and species groups

The individuals representing the putative new species from the northern mountains were distinguished in all genetic analyses from all the remaining known taxa from the Cameroon radiation. Phylogenetically, the two populations from the Gotel Mts. and Tchabal Mbabo were found to have the sister relationship, and are moderately divergent in the mitochondrial *12S–16S* fragment (5.0% uncorrected *p*-distance; in *16S* 1.2%). No shared haplotypes were found in phased *RAG1* between the two populations. Considering also the high-montane distributions (at around 2,000 m a.s.l.) of these small-sized puddle frogs and limited dispersal possibilities between the mountain ridges (separated by valleys/plateaus with elevations at maximum around 1,300 m a.s.l. with completely different habitats—relatively dry savanna), the two populations from the Gotel Mts. and Tchabal Mbabo are below described as two new species. The two new species represent a novel species group named below. The two small-sized lowland species, *P. horsti* and *P. ruthbeateae*, form a distinct clade within the Cameroon radiation, thus, we establish a new species group for them; the *P. ruthbeateae* species group. As *P. cricogaster* is consistently inferred as the sister taxon to the *P. steindachneri* complex, which includes *P. jimzimkusi* and *P. njiomock*, we treat all these taxa as members of the *P. steindachneri* species group. *Phrynobatrachus batesii* consistently clustered within the clade containing the *P. werneri* species group, and considering also the unique insertion in *16S* present in these species (4–7 bp; *P. batesii* 7 bp), we include *P. batesii* as a member of the *P. werneri* species group. The phylogenetic position of *P. chukuchuku* remains uncertain, therefore, we treat this species as a distinct evolutionary lineage not assigned to any of the previous species groups.

### Descriptions of two new species

Family Phrynobatrachidae Laurent, 1941

Genus *Phrynobatrachus* Günther, 1862

Species group *Phrynobatrachus arcanus—*new species group

***Phrynobatrachus arcanus* sp. nov.**

**Figure 3:**
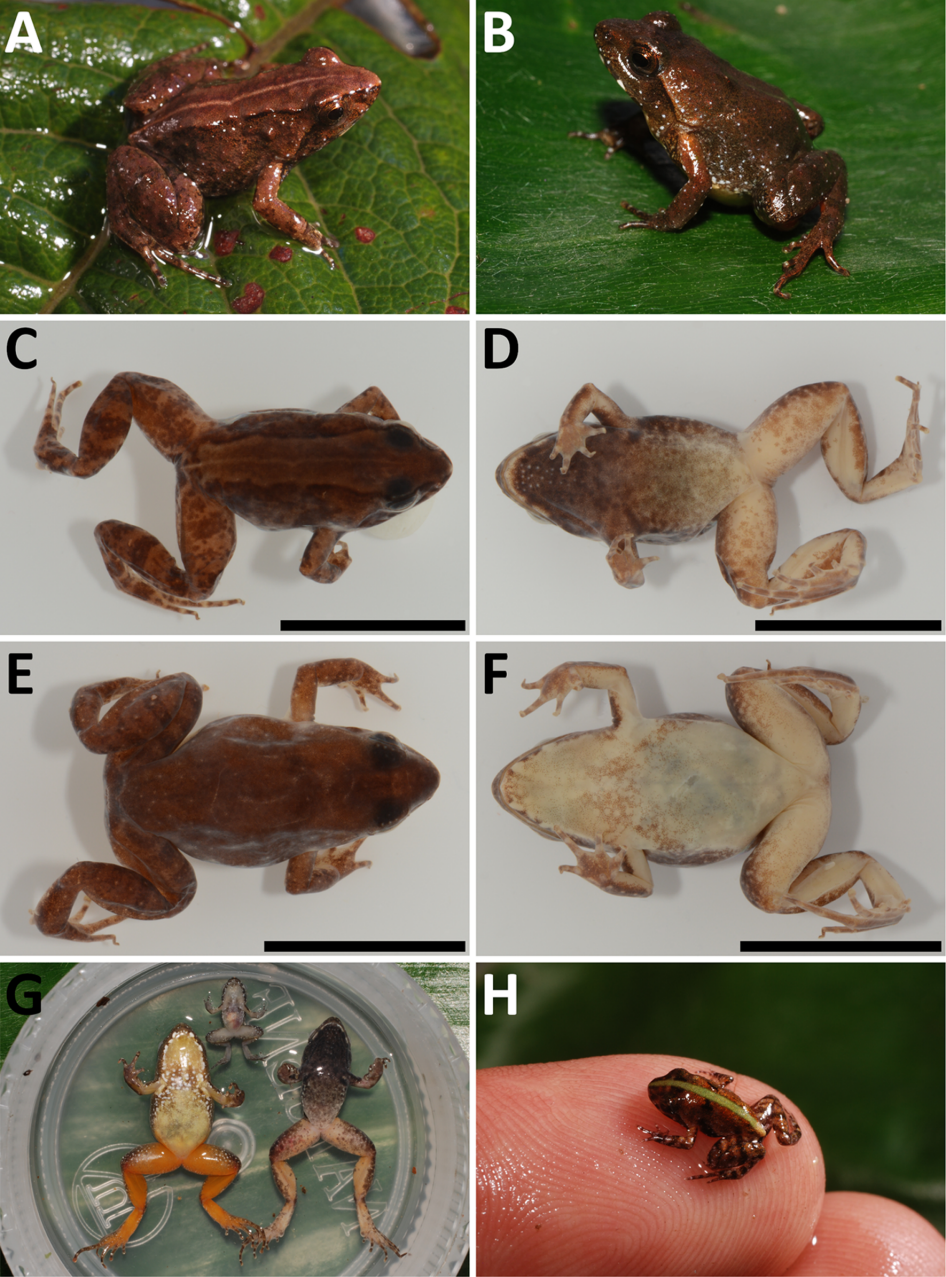
*Phrynobatrachus arcanus* sp. nov. (A) Holotype (NMP-P6V 74603/1, adult male) in life, and (C and D) in preservative in dorsal and ventral views. (B) Allotype (NMP-P6V 74603/2, adult female) in life, and (E and F) in preservative in dorsal and ventral views. (G) Comparison of ventral sides of holotype, allotype and a metamorph (NMP-P6V 74603/7), and (H) the same metamorph in life. Scale = 10 mm.

**Figure 4:**
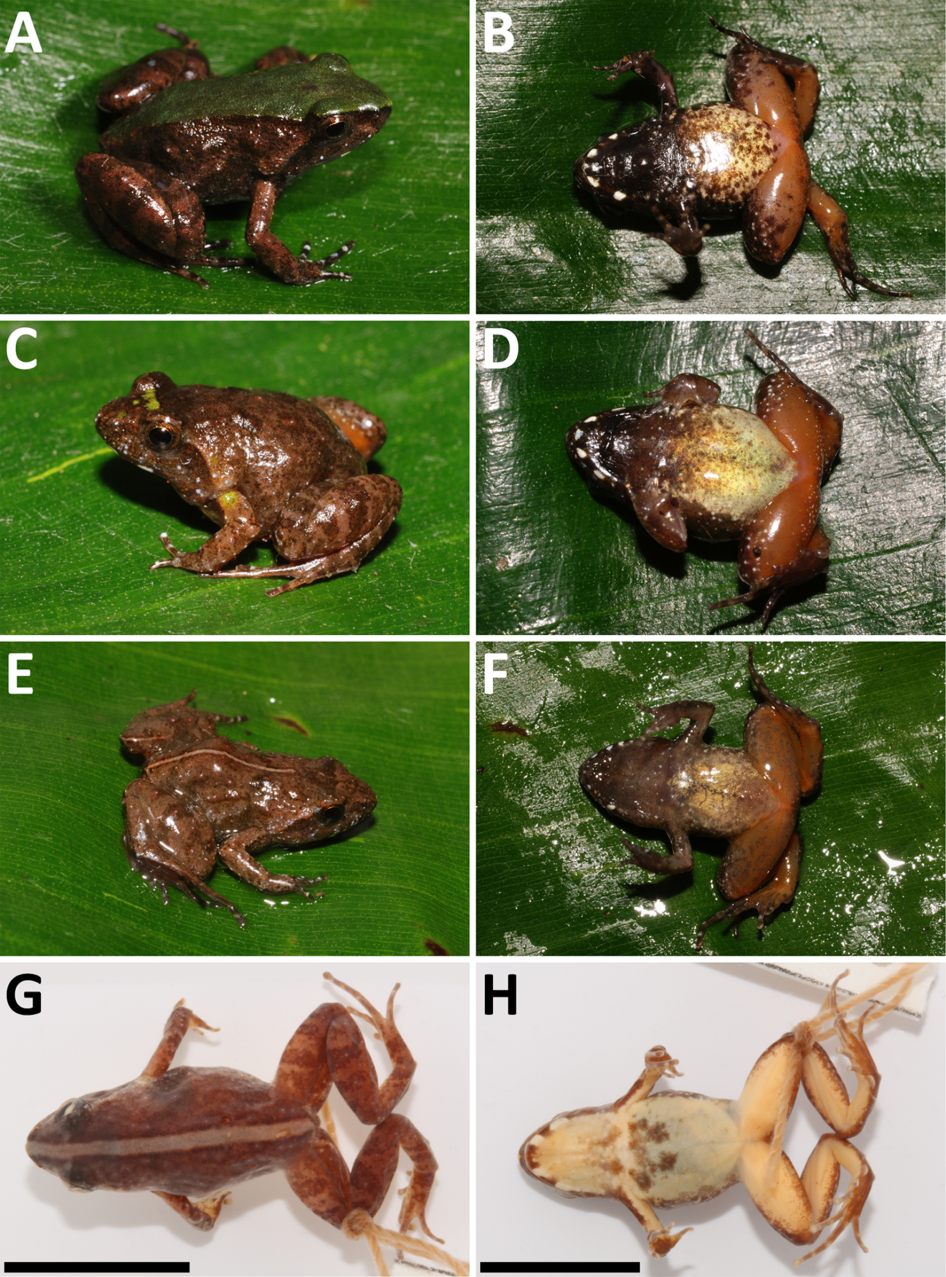
*Phrynobatrachus arcanus* sp. nov. variation in paratypes. Males: (A and B) NMP-P6V 74603/4, (C and D) NMP-P6V 74603/5, (E and F) NMP-P6V 74604; and female: (G and H) ZFMK 47956. Scale = 10 mm.

**Figure 5:**
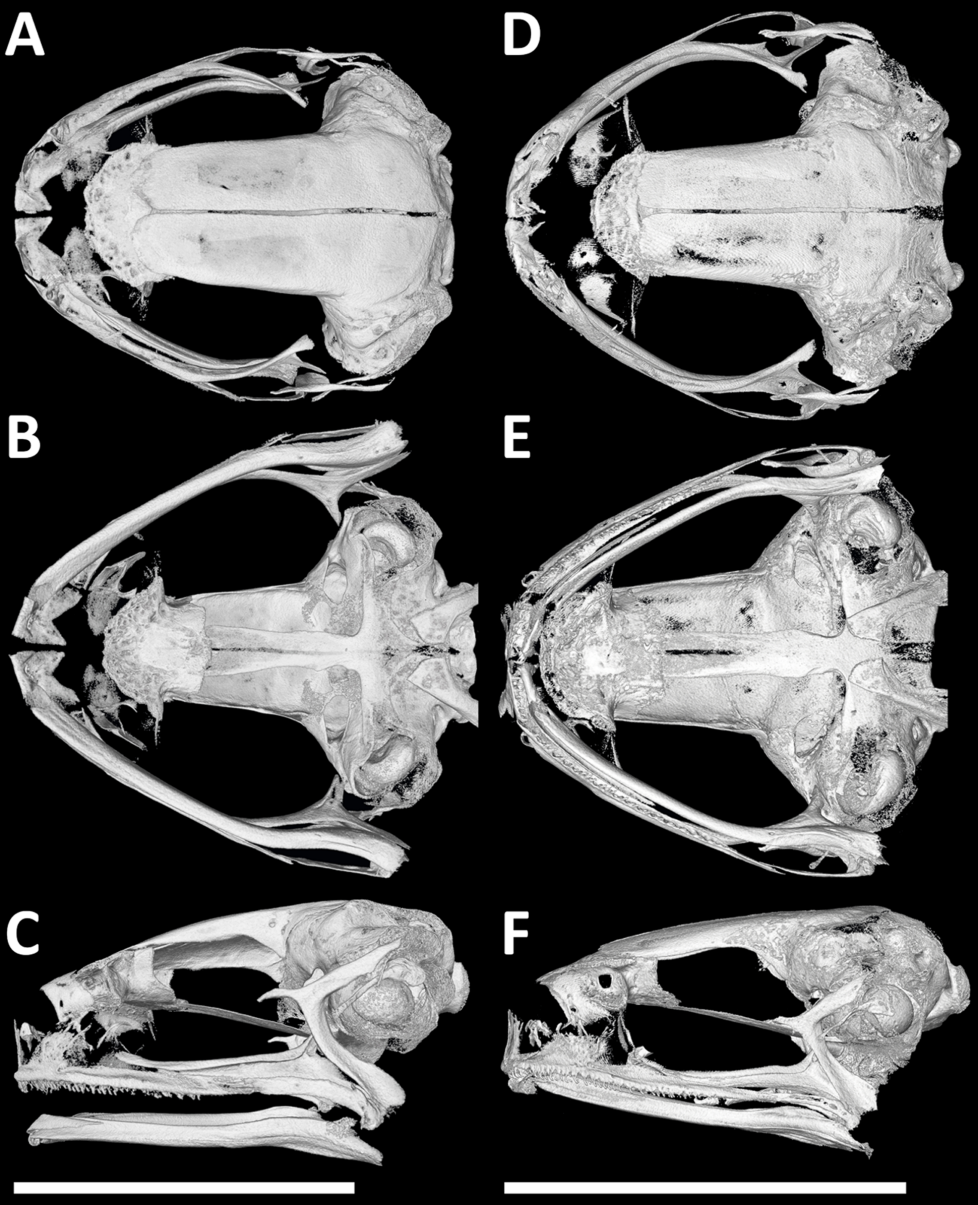
Cranial morphology (μCT scans) of males in dorsal, ventral and lateral views. (A–C) *P. arcanus* sp. nov. (holotype), and (D–F) *P. mbabo* sp. nov. (allotype). Scale = 5 mm.

**Figure 6:**
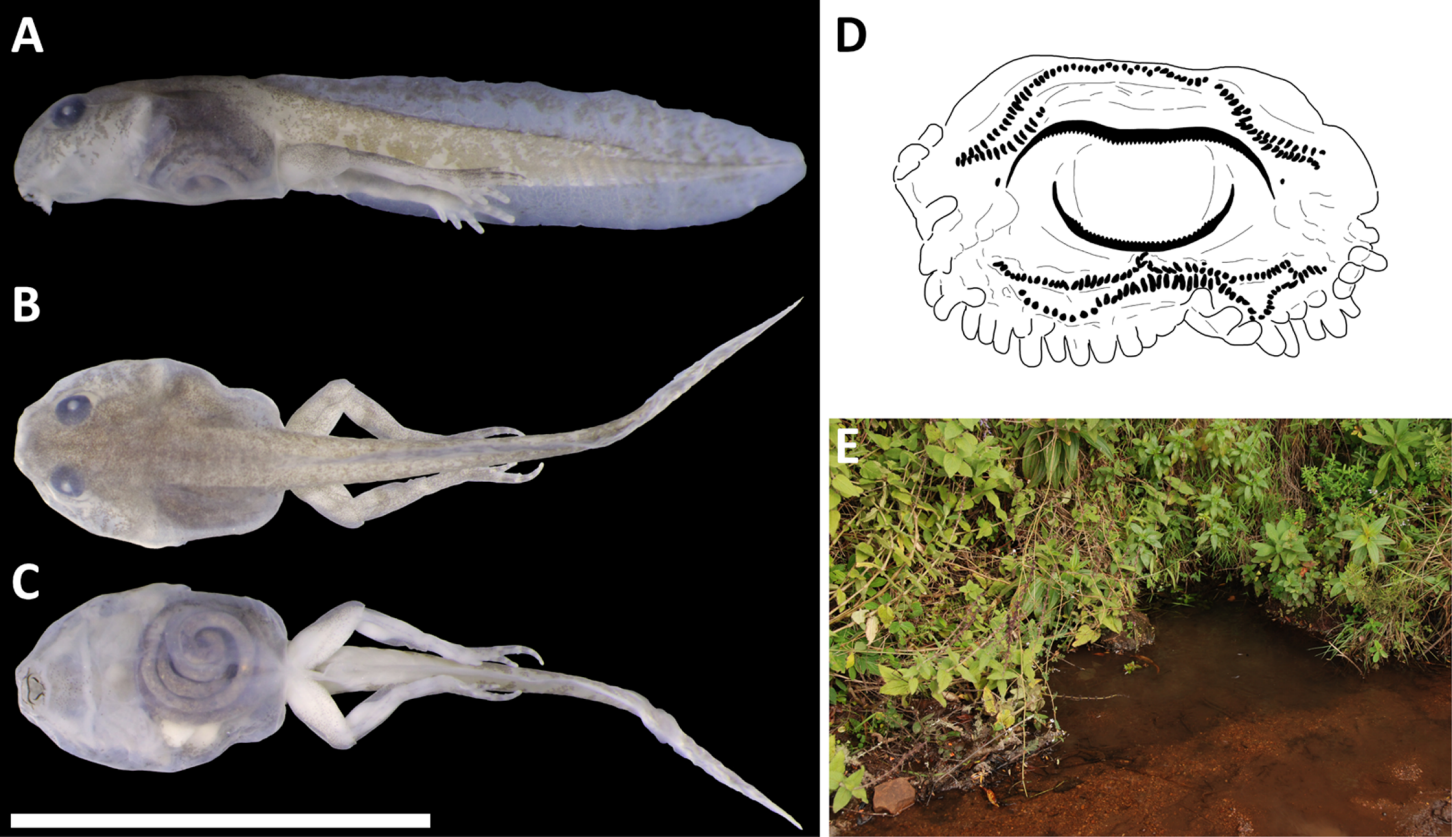
Tadpole of *P. arcanus* sp. nov. and habitat. (A–D) NMP-P6V 75866/A in lateral, dorsal and ventral views (scale = 10 mm), and sketch of the oral disc. (E) The pond at the type locality, where tadpoles were collected.

**Figure 7:**
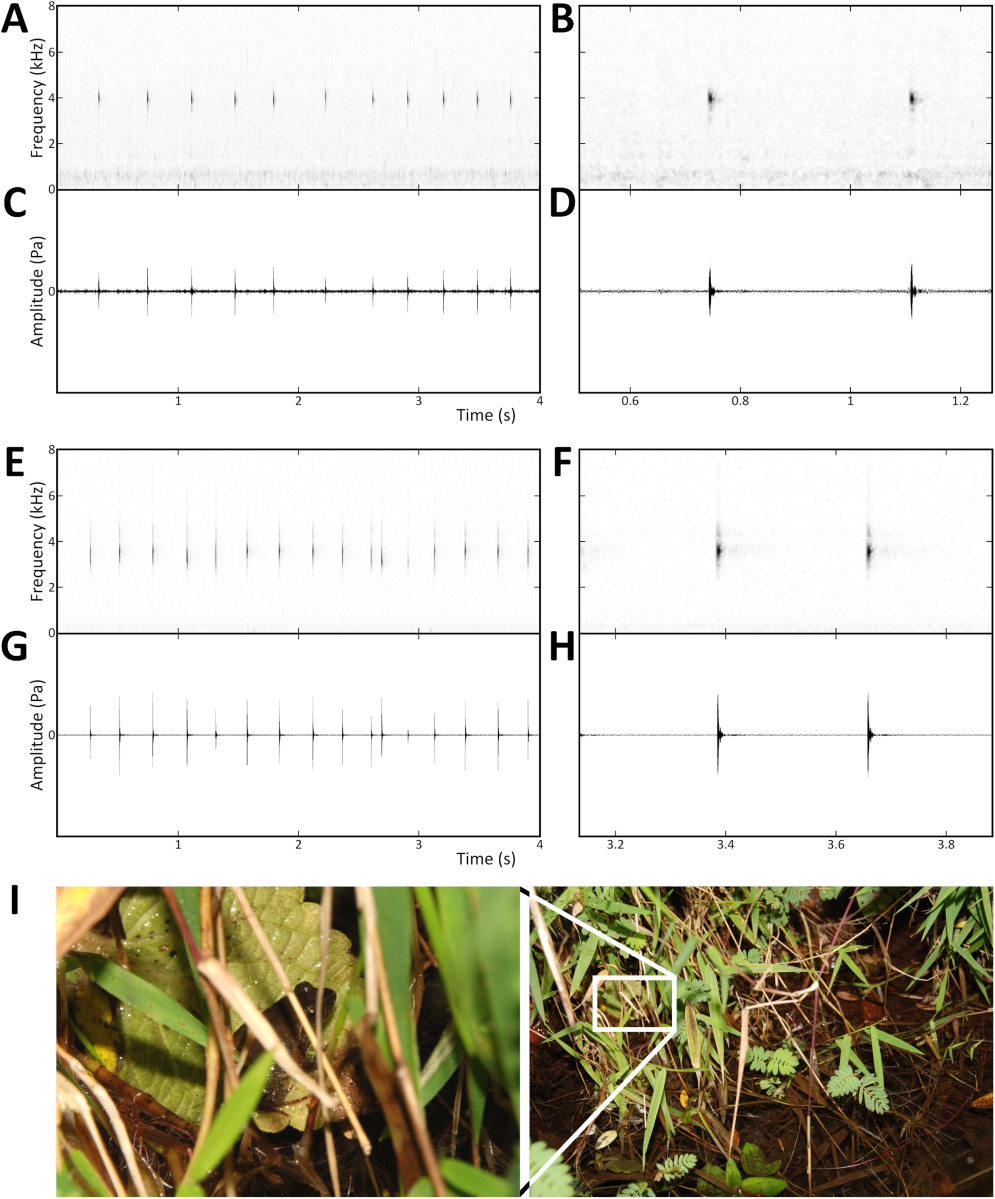
Advertisement calls of *P. arcanus* sp. nov. and *P. mbabo* sp. nov. and microhabitat. Spectrograms (A and B, E and F) above oscillograms (C and D, G and H): (A–D) *P. arcanus* sp. nov., and (E–H) *P. mbabo* sp. nov. (I) Male of *P. arcanus* sp. nov. in the calling position in the inset of the photo of microhabitat at the type locality.

ZooBank registration: urn:lsid:zoobank.org:act:C9921DC5-52CE-4CDF-A0A5-AE77D044299A

(Figs. 3–7; Tables 1–4)

Synonymy:

*Phrynobatrachus* sp.—Böhme & Nikolaus, 1989: 29 (Remark: “*Phrynobatrachus* cf. *werneri*” corresponds to *P. steindachneri* based on our morphological investigations).

*Common name:* Hidden Puddle Frog.

**Table 3:** Morphometry of adult *P. arcanus* sp. nov. For measurement abbreviations see “Materials and Methods”, SD, standard deviation; means in bold. Original data in Table S1.

	*P. arcanus* sp. nov. —males (*n* = 7)					*P. arcanus* sp. nov.—females (*n* = 4)				
(mm)	Holotype	Mean	SD	Min	Max	Allotype	Mean	SD	Min	Max
SVL	13.8	**14.5**	1.1	13.1	17.0	14.8	**16.0**	0.8	14.8	16.9
SUL	13.6	**14.2**	1.0	13.0	16.3	14.3	**15.6**	0.8	14.3	16.5
HW	5.2	**5.4**	0.2	5.0	5.6	5.8	**5.6**	0.1	5.5	5.8
HDL	4.9	**5.0**	0.2	4.8	5.3	5.1	**5.4**	0.2	5.1	5.7
TD	1.1	**1.0**	0.1	0.8	1.1	1.0	**0.9**	0.1	0.8	1.1
ED	1.8	**2.0**	0.1	1.8	2.2	2.0	**2.1**	0.1	1.9	2.2
IOD	1.2	**1.7**	0.2	1.2	1.9	1.5	**1.7**	0.1	1.5	1.8
EAD	2.7	**2.8**	0.2	2.5	3.0	2.9	**2.8**	0.1	2.8	2.9
EPD	4.5	**4.4**	0.2	4.1	4.7	4.1	**4.7**	0.4	4.1	5.0
IND	2.3	**2.2**	0.1	1.9	2.4	2.0	**2.1**	0.1	2.0	2.2
SL	1.9	**2.0**	0.2	1.8	2.5	2.5	**2.2**	0.2	1.9	2.5
SNL	0.6	**0.8**	0.2	0.6	1.1	1.0	**1.1**	0.1	1.0	1.3
ENL	1.4	**1.2**	0.2	1.0	1.7	1.5	**1.1**	0.3	0.9	1.5
HL	2.8	**2.4**	0.3	2.1	3.0	2.4	**2.8**	0.3	2.4	3.0
RL	3.4	**2.9**	0.2	2.6	3.4	3.1	**2.8**	0.2	2.7	3.1
MD1	1.5	**1.0**	0.2	0.8	1.5	1.0	**1.3**	0.2	0.9	1.6
MD2	1.5	**1.2**	0.1	1.1	1.5	1.2	**1.4**	0.2	1.2	1.7
MD3	2.5	**1.9**	0.4	1.3	2.5	1.9	**2.3**	0.2	1.9	2.5
MD4	1.2	**1.2**	0.1	1.0	1.4	1.0	**1.2**	0.2	1.0	1.4
PD1	1.3	**0.8**	0.2	0.5	1.3	0.8	**0.9**	0.2	0.7	1.2
PD2	1.8	**1.3**	0.3	1.0	1.8	1.2	**1.5**	0.2	1.2	1.9
PD3	2.6	**2.4**	0.3	2.1	2.9	2.3	**2.8**	0.4	2.3	3.4
PD4	4.5	**4.1**	0.3	3.7	4.7	4.3	**4.7**	0.2	4.3	5.0
PD5	2.1	**2.1**	0.1	2.0	2.2	2.1	**2.4**	0.3	2.1	2.9
FL	6.6	**6.7**	0.5	6.0	7.6	7.2	**7.1**	0.4	6.5	7.5
TL	7.1	**7.0**	0.4	6.3	7.6	7.3	**6.7**	0.6	6.1	7.3
FTL	8.8	**7.3**	0.7	6.6	8.8	7.0	**7.1**	0.2	6.8	7.4
IMTL	0.8	**0.9**	0.1	0.8	1.1	0.8	**0.8**	0.0	0.7	0.8
OMTL	0.7	**0.5**	0.1	0.3	0.7	0.5	**0.4**	0.1	0.4	0.5

**Table 4:** Measurements (in mm) and body indices of tadpoles of *P. arcanus* sp. nov. (NMP-P6V 75866). For measurement abbreviations see “Materials and Methods”. Means in bold.

Specimen	A	B	C	Mean		A	B	C	Mean
stage	39	36	36			39	36	36	
Ltot	18.9	15.8^*^	19.9	**18.2**	TAL/Ltot	0.68	N/A	0.69	**0.69**
BL	6.0	5.9	6.3	**6.1**	BL/Ltot	0.32	0.37	0.32	**0.34**
TAL	12.9	9.9^*^	13.7	**12.1**	TAL/BL	2.10	N/A	2.20	**2.15**
BH	2.7	2.6	2.5	**2.6**	BH/BL	0.45	0.43	0.39	**0.43**
BW	4.2	3.8	4.0	**4.0**	BW/BL	0.70	0.65	0.64	**0.66**
TW	1.4	1.2	1.3	**1.3**	SSD/BL	0.44	0.51	0.39	**0.45**
THB	2.0	1.4	1.5	**1.6**	SL/BL	0.18	0.18	0.16	**0.17**
DFH	1.3	1.4	1.2	**1.3**	ED/BL	0.14	0.12	0.12	**0.13**
VFH	1.1	1.1	1.1	**1.1**	ODW/BW	0.30	0.31	0.33	**0.32**
THM	3.4	3.7	3.3	**3.4**	TW/BW	0.34	0.32	0.34	**0.33**
ED	0.8	0.7	0.8	**0.8**	TAL/THM	3.79	N/A	4.15	**3.97**
ECD	0.9	1.0	1.0	**1.0**	THB/BH	0.74	0.53	0.62	**0.63**
SED	1.5	1.6	1.5	**1.5**	THM/BH	1.24	1.43	1.33	**1.33**
ODW	1.3	1.2	1.3	**1.3**	DFH/VFH	1.21	1.28	1.08	**1.19**
SL	1.1	1.1	1.0	**1.1**	DFH/THM	0.38	0.39	0.35	**0.38**
SSD	2.6	3.0	2.5	**2.7**	VFH/THM	0.32	0.30	0.33	**0.31**

**Note:**

* Missing tail tip.

*Etymology:* The species epithet is derived from the Latin adjective “*arcanus*,” meaning “hidden” or “secret”. It refers to the fact that this species is difficult to find with only seven adults discovered in herbaceous vegetation (all collected in 2009) during three visits to the type locality (2009, two in 2016). Similarly, G. Nikolaus collected only four specimens among 54 individuals of *Phrynobatrachus* in 1988 (the others being *P. steindachneri*—our investigation; Böhme & Nikolaus, 1989). Moreover, the failure to find this species in 2016 despite intensive searches may be related to a population decline, which is known to have occurred in *Phrynobatrachus* at different sites in the CVL (Hirschfeld et al., 2016; Doherty-Bone & Gvoždík, 2017; Tchassem et al., 2019).

*Holotype:* NMP-P6V 74603/1, adult male, Nigeria–Cameroon border area, Gotel Mountains, Mount Gangirwal (= Chappal Waddi or Cabbal Wade), site 2 (pond in small stream surrounded by shrubs on grassland slope), 7.0398°N, 11.7068°E, 2,250 m, 5.XI.2009, leg. V. Gvoždík.

*Paratypes:* NMP-P6V 74603/2, adult female (allotype), NMP-P6V 74603/3-6, four adult males, same collection data as holotype; NMP-P6V 74604, adult male, Nigeria–Cameroon border area, Gotel Mountains, Mount Gangirwal (= Chappal Waddi or Cabbal Wade), site 1 (stream in escarpment forest), 7.0307°N, 11.7021°E, 1,940 m, 4.XI.2009, leg. V. Gvoždík; ZFMK 47956 (adult female), ZFMK 47958 (adult male), Nigeria, Gotel Mountains, Mount Gangirwal, 7.03°N, 11.70°E, 2,300 m, III.1988, leg. G. Nikolaus.

*Additional material:* ZFMK 47955, 47957 (females), same collection data as the ZFMK paratypes. NMP-P6V 74603/7-8, juveniles, same collection data as holotype (NMP-P6V 74603/8 collected on 6.XI.2009). Larvae: NMP-P6V 75866/A–C, vg09-173 (field number), four tadpoles (three in formalin—morphological vouchers, one in ethanol—genetic voucher), stages 36 and 39 (Gosner, 1960), same collection data as holotype.

*Definition:* Small-sized *Phrynobatrachus* (SVL = 13.1–17.0 mm); small asperities as a medial stripe in gular region in males, arranged into a rectangle widening in anterior direction, number and density of the asperities increases anteriorly (Fig. 3D); distinct white spots on ventral side of lower jaw; dark brown or gray-black gular and pectoral regions in males; posterior venter coloration in males lighter with dark spots extending from chest; yellowish venter with white spots on brown background on chest in females; smooth light gular region in females with same coloration as posterior venter; vividly colored ventral side of hind legs in both sexes, more vivid (dark orange) in females, yellowish-pink with orange tint in males; small white dots/asperities on body sides and front sides of thighs; naris small with raised narial ring, positioned usually closer to snout tip than to eyes (ENL/SL = 0.5–0.7); dark brown tympanum inconspicuous covered by skin; supratympanic fold ranging from the posterior corner of the eye to the shoulder, lighter colored than dark tympanic region; canthus rostralis short and rounded; loreal region faintly concave; eyelid cornicles absent; pupil round; maxillary and premaxillary teeth present; vomerine teeth absent; thigh and shank of similar length; webbing on front limbs absent; pedal webbing rudimentary (extending to first proximal phalanx, four phalanges free of webbing); tarsal tubercle present; small outer metatarsal tubercle; relatively robust, protruding inner metatarsal tubercle of similar length like the first toe.

*Differential diagnosis (adults): Phrynobatrachus arcanus* can be distinguished from other montane species of the Cameroon radiation by the combination of the following features: by smaller size (SVL = 13.1–17.0 mm) from majority of other species, see values below; from *P. chukuchuku* (SVL = 14.8–19.2 mm) by position of asperities in gular region in a medial stripe (throughout gular region in *P. chukuchuku*), and lighter coloration of ventral side of body in males (Zimkus, 2009); from *P. werneri* (SVL = 17.4–23.0 mm) and *P. manengoubensis* (SVL = 14.1–19.6 mm) by less pronounced skin folds in gular region of males, vivid orange or pinkish ventral coloration of hind limbs, and absence of yellow coloration of posterior venter (Nieden, 1910; Angel, 1940; Perret, 1966; Blackburn & Rödel, 2011); from *P. schioetzi* (SVL = 22.6–27.7 mm) by less pronounced skin folds in gular region, position of asperities in a medial stripe in gular region (at posterolateral margins of lower jaw in *P. schioetzi*), and absence of light stripes along supratympanic fold and on lateral sides (Blackburn & Rödel, 2011); from *P. danko* (SVL = 19.6–21.8 mm) by position of asperities in gular region in a medial stripe (along the edge of lower jaw in *P. danko*), and lighter coloration of gular region in males (Blackburn, 2010); from *P. cricogaster* (SVL = 20.0–32.0 mm) by smoother dorsal skin, presence of only one tarsal tubercule, and absence of typical bullseye coloration of venter (Perret, 1966); and from the *P. steindachneri* species complex (SVL = 26.0–36.0 mm; *P. jimzimkusi*, *P. njiomock*, *P. steindachneri*—syntopic species, see Fig. S2) by substantially smaller size, less developed pedal webbing, less conspicuous tympanum, and absence of small spines on the plantar surface and ventral side of tarsus (Nieden, 1910; Blackburn & Rödel, 2011; Zimkus & Gvoždík, 2013; T. Nečas & V. Gvoždík, 2019, unpublished data). For differential diagnosis of tadpoles see below.

*Description of holotype (external morphology):* Adult male (Figs. 3A, 3C, 3D and 3G), snout-vent length of 13.8 mm; oval body with slender limbs; head slightly wider than long (HW/HDL = 1.06); canthus rostralis short and rounded; distance between naris is larger than between upper eyelids (IND/IOD = 1.92); tympanum indistinct with diameter approximately two-thirds of diameter of eye (TD/ED = 0.61); supratympanic fold present; dorsal chevron-shaped glands small, slightly protruding; palmar and thenar tubercles present; fingers with round subarticular tubercles; no webbing present between fingers; finger tips slightly bulbous; webbing between toes extremely rudimentary (four phalanges free of webbing on PD4); one tarsal and one small heel tubercle present; inner metatarsal tubercle and outer metatarsal tubercle present, the latter smaller (OMTL/IMTL = 0.88); smooth stripe of skin on the dorsal side delimited by rows of asperities on both sides; lateral side covered in asperities of different sizes; ventral side smooth except stripe of small lightly-colored asperities in a medial stripe on throat tapering in direction to chest.

*Measurements of holotype:* See Table 3.

*Coloration of holotype in life* (Figs. 3A and 3G): Dorsal coloration dark brown; from snout tip to back edge of urostyle can be seen thick stripe of light brown color bounded by thin black line on each side; thin light vertebral line is also present that ends near cloaca and branches to back sides of thighs; whole dorsum is irregularly covered in small spots of orange and more rarely white color; tympanum is covered by dark brown spot; lateral sides display sparsely placed white spots near hind limbs; dorsal coloration of limbs is slightly darker than of dorsum; manual digits on dorsal side bear dark coloration in joints; these stripes can be seen on back limbs too, but are not only on joints. Ventral coloration of gular region is dark brown to black with line of white asperities tapering in the direction of the venter; along edges of lower jaw are symmetrically placed white spots; dark gular coloration extends to anterior side of arms and forearms, where it displays white spots; ventral side of arms and forearms is pinkish; venter light yellow to cream in the direction of the chest more brown; yellow venter is bound by black spots; black stripe over chest separates yellow belly from dark throat; ventral side of hind limbs and groin pinkish with orange tint; front side of thighs with small white dots on larger brown to gray spots.

*Coloration of holotype in preservative* (Figs. 3C and 3D): Dorsum dark brown; thick brown stripe extends from snout tip to posterior edge of urostyle; this brown stripe is bounded by thin black line on each side; thin light line along vertebra is also present; sides brown-gray with dark spots; dorsal side of back limbs bears brown spots on underlying yellow on thighs and light brown on calves. Gular region gray to black; this coloration extends to front sides of front limbs; edge of lower jaw fringed by white spots; group of white (or colorless) asperities is in a medial stripe on throat, creating wedge-like shape in direction to chest; groin, ventral sides of thighs closer to body, calves and tarsa are light; venter light, covered in brown spots; palms and feet are light gray.

*Morphological variation:* Four specimens (one male, three females) were collected in 1988 (Böhme & Nikolaus, 1989; as *Phrynobatrachus* sp.), and six males, one female, two juveniles and four tadpoles in 2009. Body shape is relatively consistent among the individuals; descriptive morphometrics are provided in Table 3; the full data is provided in Table S1. Snout-vent length in males 13.1–17.0 mm (*n* = 7), 14.8–16.9 mm in females (*n* = 4), and 7.4–8.7 mm in fresh metamorphs (*n* = 2). Dorsal chevron-shaped glands mostly indistinct, especially in individuals with wide dorsal medial stripe where they align with its darkly colored margin. Loreal region lightly colored. White and orange spots may be present. Tympanum and area between eye and nostril darker than loreal region. Light or even green interorbital band might be present in individuals that do not have a wide monochromatic band on their dorsum. A pair of dark spots placed laterally on each side of the dorsal glands may be present. A thinner and lightly colored stripe along the body axis may be present. Iris mostly copper-colored. Flanks sparsely beset with small white spots. Venter in females is usually uniformly yellow. The coloration of venter in males ranges from uniformly yellow in its posterior part and mottled in anterior to gray color. Throat in males uniformly black with coloration transitioning to dark brown that extends to the anterior portion of venter and in some degree to flanks. The female gular region is usually yellow with white spots in the posterior part with underlying brown color that extend onto flanks. Both sexes have white spots along the margins of lower jaw. The ventral coloration of hind limbs in males ranges from flesh pink to orange. However, the thigh coloration in females has much more vivid orange color. Both sexes also display small white spots on anterior sides of thighs (See Fig. 4).

*Cranial osteology:* Male holotype (Figs. 5A–5C) and female paratype (Figs. S3A–S3C) do not differ in general cranial morphology: The ventromedial portion of sphenethmoid fuzed; palatines present but reduced to thin bones only; laterally positioned vomers reduced and lacking teeth-bearing dentigerous processes; maxillary and premaxillary teeth present; alary process of premaxilla inclined laterally; anterior rami of pterygoids separated from maxilla; medial rami of pterygoids reduced in length; no mandibular teeth-like structures present; tip of cultriform process of parasphenoid serrated; body of cultriform process narrowed at its base; parasphenoid alae moderately long and oriented perpendicularly to anteroposterior body axis; nasals triangular and not overlapping sphenethmoid, and not in contact medially; otic foramen large; medial and lateral anterior margins of frontoparietals extend to the same level; frontoparietals slightly wider posteriorly than anteriorly; ventral ramus of squamosal slightly curved in posterior direction and relatively long; zygomatic rami of squamosal wider and longer than otic rami; anterior processes of quadratojugals laterally overlapped, but not in contact with maxilla; columella relatively robust.

*Tadpole description* (Figs. 6A–6D): Body is ovoid-shaped with rounded snout; body reaches the widest point near posterior end of eyes; eyes are placed dorso-laterally, where eye diameter constitutes 13% of body length; tail constitutes half of entire length of tadpole and has moderately developed fin; dorsal fin begins at the tail base; ventral fin begins at the ventral end of the body, is thinner than the base of tail and slightly curved; tail tip is rounded (possibly not naturally); dorsal fin is higher than ventral fin (DFH/VFH = 1.19); maximal tail height is higher than height of the body (THM/BH = 1.33); width of tail is 0.33 of body width; maximal height of tail is 0.63 of body height; ventral tube dextral; spiraculum on the left side, slightly anterior from the body center (SSD/BL = 0.45); length of spiraculum is 0.17 of the body length; mouth opens anterio-ventrally; width of oral disc is 0.32 of body width; lower labium neighbors one row of slender papillae with rounded tips extending up to lateral position; upper labium without any papillae; filamentous papillae absent; tooth (keratodont) formula 1/1 + 1//2; keratinized parts of jaw sheath thin and finely serrated; upper jaw has shape of wide letter M, while lower of letter V or U. Tail and body have dark color with spots on dorsal side; these spots are less frequent in the direction to tail tip; ventral side not pigmented; fins transparent with occasional dark spot on the dorsal fin; spiraculum and ventral tube transparent; intestine visible. For measurements, see Table 4.

*Morphological comparison of tadpoles:* Larvae/tadpoles of only three species of *Phrynobatrachus* of the Cameroon radiation were described (Pfalzgraff et al., 2015): *P. chukuchuku*, *P. jimzimkusi* (*P. steindachneri* species group), and *P. manengoubensis* (*P. werneri* species group). All are very similar to each other in their ovoid to oval body shape and cryptic coloration. Total length of similar stages (Gosner stages 36–41) is known only for *P. arcanus* (Ltot = 15.8–19.9 mm) and *P. chukuchuku* (Ltot = 19.1–19.5 mm). The two species with a similar adult size (SVL < 20 mm) have also similar size of larvae and similar ratio of body and tail lengths (BL/TAL = 0.51 vs. 0.53). Tadpoles of *P. arcanus* can be distinguished from the three other known species (Pfalzgraff et al., 2015) by: a number of tooth/keratodont rows (two posterior rows in *P. arcanus* and *P. manengoubensis* vs. three posterior rows in *P. chukuchuku* and *P. jimzimkusi*; two anterior rows in *P. arcanus* vs. one anterior row in *P. manengoubensis*); larger relative oral disc width (ODW/BW = 0.3 in *P. arcanus* vs. 0.2 in *P. chukuchuku*, *P. jimzimkusi* and *P. manengoubensis*); and larger relative maximal tail height (THM/BH = 1.3 in *P. arcanus* vs. 0.9 in *P. chukuchuku*, and 1.0 in *P. jimzimkusi* and *P. manengoubensis*). Channing, Rödel & Channing (2012) described and figured a tadpole of “*P. steindachneri*”, which was later reidentified as *P. jimzimkusi* based on the locality data (Pfalzgraff et al., 2015). The descriptions by the two author teams differ in several substantial characters, that is, presence of filamentous papillae described by the first authors vs. absence by the latter authors. If we consider the fact that the tadpole was not genotyped by the first authors, and that filamentous papillae are absent in all so far described tadpoles of the Cameroon radiation *Phrynobatrachus*, the description of “*P. steindachneri*” (Channing, Rödel & Channing, 2012) must be taken with a caution as it might be a description of another species. For alternative discussion as well as more comparative data within the genus, see Pfalzgraff et al. (2015).

*Genetics:* In the 510 bp long 16S rRNA fragment, two haplotypes differing by two substitutions were found in three and three specimens, respectively (six sequenced specimens in total). Average genetic distances are shown in Table 2. Except the new puddle frog from Tchabal Mbabo, described below, all other species from the Cameroon radiation differ by 3.3% (*P. chukuchuku*) to 5.6% (*P. manengoubensis*). The new species (sister species) from Tchabal Mbabo differs in 1.2% in *16S*, and 5.0% in the whole *12S–16S* fragment (as used in the phylogenetic analyses). The sister species from Tchabal Mbabo does not share haplotypes of the nuclear *RAG1* with *P. arcanus*. In the latter species, a high heterozygosity was observed in *RAG1* with eight inferred haplotypes in the six specimens, and the haplotypes are not shared with any other *Phrynobatrachus* species examined in this study.

*Advertisement call:* Clicking notes of a slightly metallic, tonal and not frequency-modulated sound, relatively slower “tick–tick–tick–tick–tick…” increasing the repetition rate during a call to “tick-tick-tick-tick-tick…” (Figs. 7A–7D). The length of the entire call varies from a couple of seconds to more than 1 min. Notes are produced in note groups, which are distinguishable by lower amplitude intensity in the beginning and end of a note group, and by the last note with a higher pitch (dominant frequency). Note groups are usually not interspersed by distinct inter-note-group intervals. At 17.5 °C, note groups (1.7 ± 0.1 s; mean ± standard deviation) consist of 6–8 notes (clicks; 7.0 ± 0.7) with each note 12.5 ± 0.1 ms long. Average note repetition rate is 4.2 ± 0.7 notes per second, repetition rate increases during a call (~ relatively high standard deviation). The dominant frequency is at 3.92 ± 0.03 kHz.

**Figure 8:**
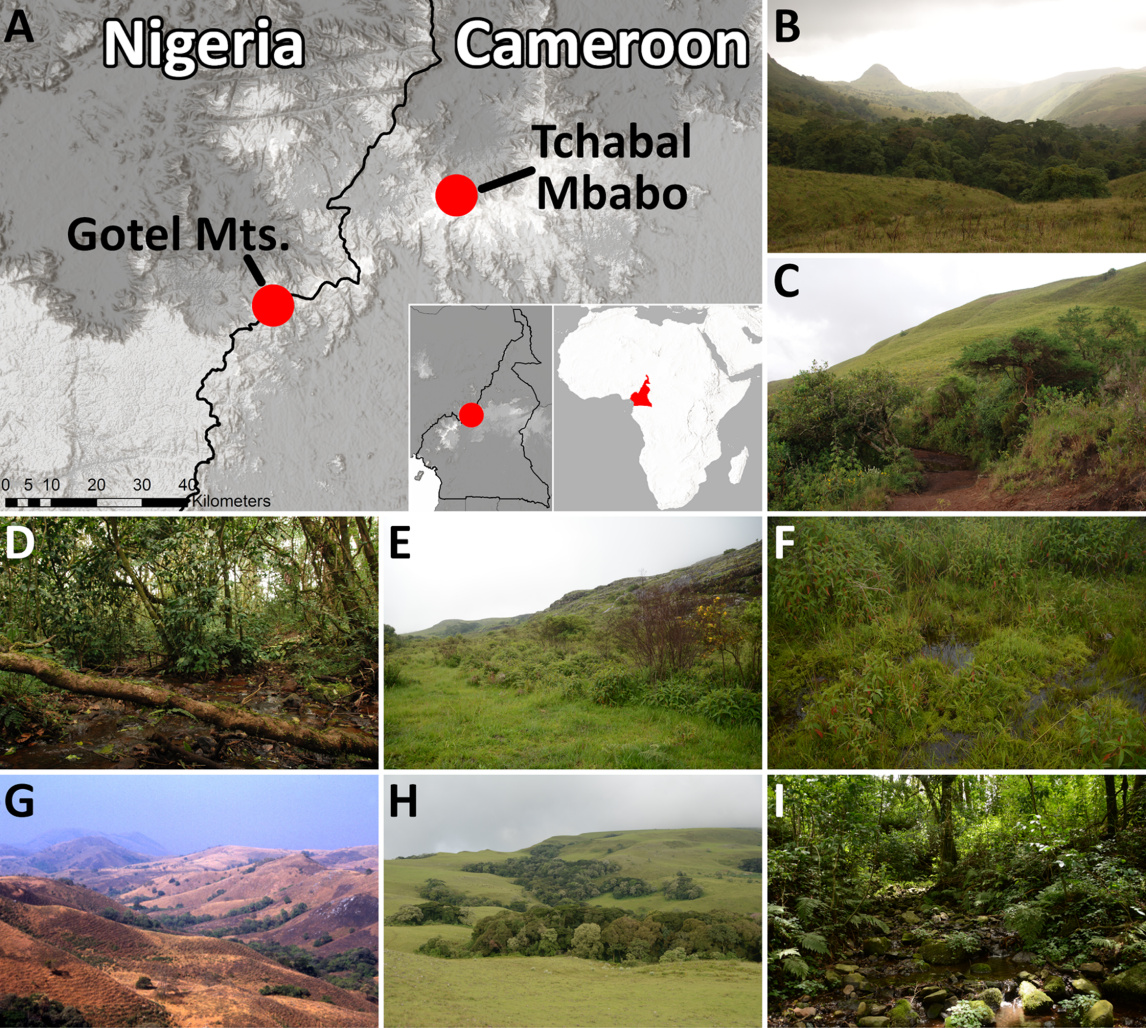
Map of the known distributions and habitats of *P. arcanus* sp. nov. and *P. mbabo* sp. nov. (A) Map of the Gotel Mountains and Tchabal Mbabo in Nigeria and Cameroon, part of the Cameroon Volcanic Line. (B–F) Habitats of *P. arcanus* sp. nov. in the Gotel Mts., and (G–I) habitats of *P. mbabo* on Mt. Tchabal Mbabo. (B) General view of a gallery forest in the Gotel Mts. at around 2,300 m a.s.l., (C) type locality of *P. arcanus* sp. nov., (D) stream in forest (locality of NMP-P6V 74604), (E and F) upland moor on the plateau near the summit of Mt. Gangirwal, where *P. arcanus* sp. nov. was probably heard in June 2016. (G) General view of gallery forests on Tchabal Mbabo at around 2,000 m a.s.l. in dry season of January 2000, (H) and in wet season of June 2016, (I) forest stream—vicinity of the type locality of *P. mbabo* sp. nov.

*Natural history:* Not much is known as only few specimens have been observed to date. Most of the specimens (NMP collection) were found during daylight, when they were actively jumping in herbaceous vegetation around a small pond on a stream outside a forest, surrounded by shrubs and montane grassland (Figs. 7C and 8C). Advertisement calls were recorded on 5 November 2009 (end of wet season) in the afternoon around 16:00. After dusk, no calling was heard or active individuals seen at the site. As only a single male was found at night near a stream in an escarpment forest (Fig. 8D) relatively near the margin of the forest (ca. 200 m), it seems that *P. arcanus* has rather diurnal activity and probably prefers open habitats, though it can be occasionally found at forested sites. Tadpoles were found in a small pond within a small stream (Fig. 6E), where males were vocalizing from partly submerged plants sitting on leaves above water surface (Fig. 7I; water temperature 17.5 °C). Not recorded, and not seen, but several individuals probably referable to this species were heard shortly before dusk (the site was quiet after the dusk) at another site (7.0489°N, 11.7079°E, 2,260 m a.s.l.) on 15 June 2016 (wet season) at 18:00, a site which might be characterized as upland moor on an open plateau near the summit (Figs. 8E and 8F). At both confirmed sites, *P. arcanus* was found syntopically with *P. steindachneri*, as also noted by Böhme & Nikolaus (1989; though, the latter was incorrectly identified as *P*. cf. *werneri*).

*Distribution:* Known only from Mt. Gangirwal, Gotel Mts., Cameroon–Nigeria border (Figs. 8A–8F), where the species was found at two sites, approximately 2 km from each other (1.1 km by air), at elevations 1,940 and 2,250 m a.s.l. A record by Böhme & Nikolaus (1989) as *Phrynobatrachus* sp. is not precisely localized but the given geographic coordinates (7.03°N, 11.70°E) and elevation (2,300 m) correspond closely to our sites.

*Threat status:* The species is of a high conservation concern. *Phrynobatrachus* is disappearing from the Cameroon Highlands (Hirschfeld et al., 2016; Doherty-Bone & Gvoždík, 2017; Tchassem et al., 2019), and it seems that this is also true for *P. arcanus*. In 2016, not a single specimen of any *Phrynobatrachus* was found in the Gotel Mts., although *P. arcanus* was possibly heard. *Phrynobatrachus arcanus* was already difficult to find in 2009, when *P. steindachneri* was still common (i.e., 20 specimens per night could be found easily). However, the ecology of *P. arcanus*, presumed preference of dense herbaceous habitat, and its small size seems to complicate a localization of this species. Considering its limited range with the limited area of occupancy (<10 km^2^) within the extent of occurrence <100 km^2^, basically a single location (two sites 1 km apart), and the presumed population decline, we strongly recommend to categorize *P. arcanus* as Critically Endangered (following the IUCN criteria; IUCN Standards & Petitions Subcommittee, 2017).

***Phrynobatrachus mbabo* sp. nov.**

**Figure 9:**
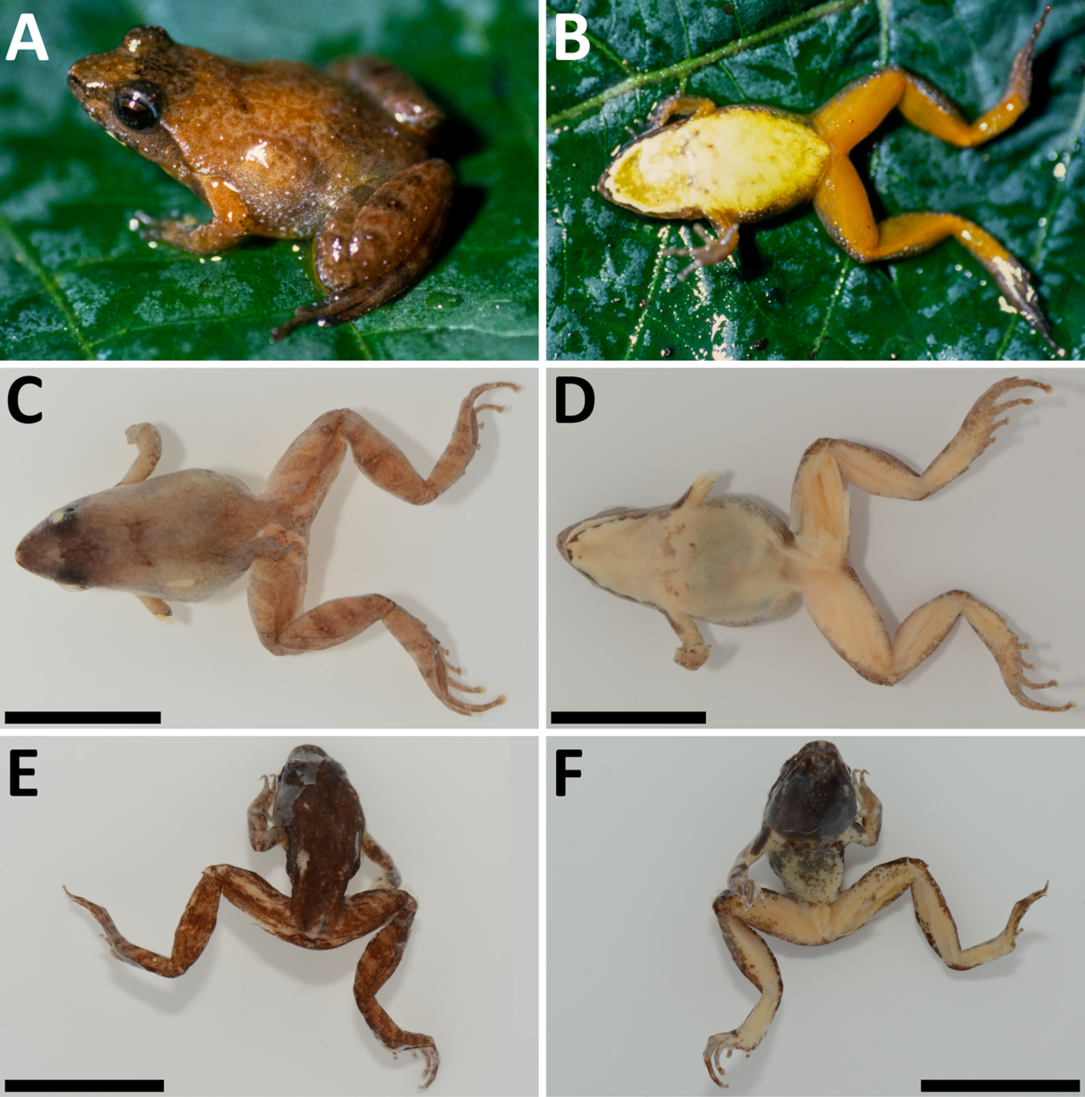
*Phrynobatrachus mbabo* sp. nov. holotype and allotype. (A–D) Holotype (ZFMK 75726, adult female) in life in dorsolateral and ventral views, and in preservative in dorsal and ventral views. (E and F) Allotype (ZFMK 75676, adult male) in preservative in dorsal and ventral views. Scale = 10 mm.

**Figure 10:**
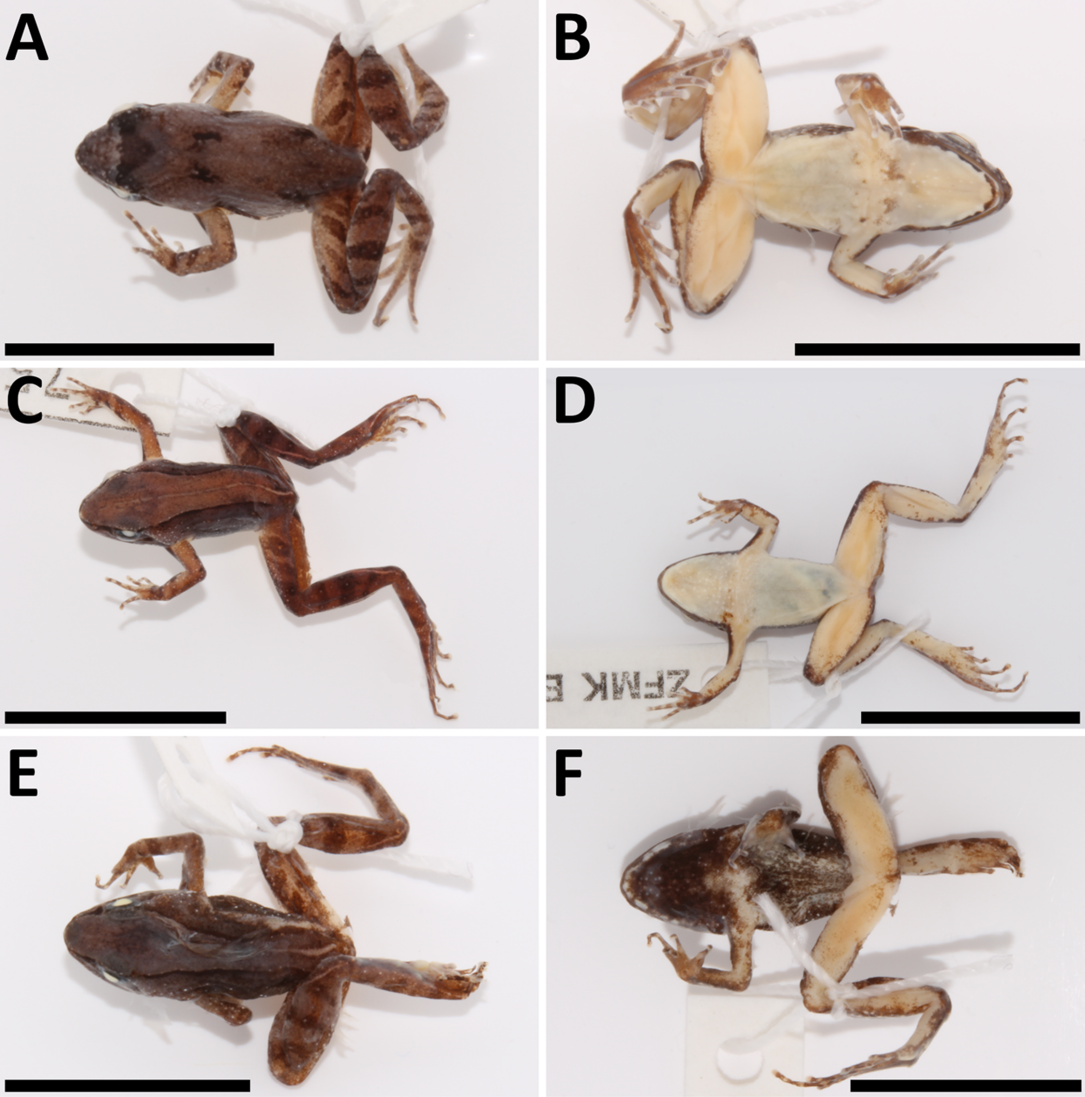
*Phrynobatrachus mbabo* sp. nov. variation shown on fixed specimens. (A and B) ZFMK 75686 (female, paratype), (C and D) ZFMK 75650 (female), (E and F) ZFMK 75683 (male). Scale = 10 mm.

ZooBank registration: urn:lsid:zoobank.org:act:422C3BB8-B16E-48ED-97FB-88D9142FEEDC

(Figs. 5, 9 and 10; Tables 1, 2 and 5)

Synonymy:

*Phrynobatrachus* sp. 5—Amiet, 1978: 6; Amiet & Goutte, 2017: 182, 266.

*Phrynobatrachus* spp. (partim)—Herrmann et al., 2007: 31 (Note: figure 7b on page 33 illustrates *P. steindachneri*).

*Common name:* Tchabal Mbabo Puddle Frog.

*Etymology:* The species epithet is a noun in apposition and refers to the mountain massif Tchabal Mbabo.

*Holotype:* ZFMK 75726, adult female, Cameroon, Adamawa Region, Tchabal Mbabo massif (southern slope), 5 km NEE of Foungoy (or Fungoi; stream in gallery forest), 7.2518°N 12.0597°E, 2060 m, 28.I.2000, leg. A. Schmitz.

*Paratypes:* ZFMK 75676, adult male (allotype); ZFMK 75677, 75686, 75728, adult females; same collection data as holotype.

*Additional material:* ZFMK 75683, adult male; ZFMK 75650, adult female; ZFMK 75729, subadult female; same collection data as holotype.

*Definition:* Small-sized *Phrynobatrachus* (SVL = 14.0–17.9 mm); gular and pectoral regions in males dark brown or gray-black with small colorless asperities arranged in a medial stripe in gular region with more conspicuous spines in the anterior part; rather smooth gular region in females with small colorless asperities arranged in the posterior part of gular region; gular region of the same color as venter with indistinctive white spots and small dark patches in pectoral region in females; yellow venter and ventral coloration of thighs light orange in females (coloration is not known in live males); naris small with raised narial ring, positioned closer to snout tip than to eyes (ENL/SL = 0.5–0.7); tympanum inconspicuous covered by darkly colored skin; supratympanic fold ranging from the posterior corner of the eye to the shoulder and lighter colored than tympanum; canthus rostralis short and rounded; loreal region slightly concave; eyelid cornicles absent; pupil round; maxillary and premaxillary teeth present; vomerine teeth absent; manual webbing absent; pedal webbing very rudimentary (extending to first proximal phalanx; four phalanges free of webbing); tarsal tubercle present; small outer metatarsal tubercle; distinct but not much protruding inner metatarsal tubercle of 0.7–1.0x length of the first toe.

*Differential diagnosis: Phrynobatrachus mbabo* (SVL = 14.0–17.9 mm) can be distinguished from other species by the combination of the same morphological features as *P. arcanus—*see above. From *P. arcanus*, *P. mbabo* can be differentiated by the following morphological disparities: osteologically, by more gracile columella and shorter ventral ramus of squamosal; in body proportions, by relatively longer hind legs [average (FL+TL+FTL)/SVL = 1.5 vs. 1.4]; in coloration of females, by less patterned pectoral region, bright yellow venter (vs. creamy), and light orange ventral side of hind legs (vs. dark orange) [coloration is not known in live males].

*Description of holotype (external morphology):* The female ZFMK 75726 (Figs. 9A–9D) was chosen as the holotype—even though the left front limb is partly missing—due to the bad condition of the only two available males. The holotype female is also the only individual of this species photographed in life. Adult female, snout-vent length 17.9 mm; oval body with slender limbs; head slightly wider than long (HW/HDL = 1.04); canthus rostralis short and rounded; distance between naris is larger than between upper eyelids (IND/IOD = 1.38); tympanum indistinct covered by skin approximately half the diameter of eye (TD/ED = 0.43); supratympanic fold present; dorsal glands small, slightly protruding; palmar tubercles present; fingers with round subarticular tubercles; no webbing present between fingers; finger tips slightly bulbous; webbing between toes extremely rudimentary (four phalanges free of webbing on PD4); one tarsal and one small heel tubercle present; outer metatarsal tubercle smaller than inner metatarsal tubercle (OMTL/IMTL = 0.63); dorsal skin smooth with sparsely scattered asperities; ventral skin smooth.

*Measurements of holotype:* See Table 5.

**Table 5:** Morphometry of adult *P. mbabo* sp. nov. For measurement abbreviations see “Materials and Methods”, SD, standard deviation; means in bold. Original data in Table S2.

	*P. mbabo* sp. nov.—males (*n* = 2)					*P. mbabo* sp. nov. —females (*n* = 5)				
(mm)	Allotype	Mean	SD	Min	Max	Holotype	Mean	SD	Min	Max
SVL	14.0	**14.3**	0.3	14.0	14.5	17.9	**16.3**	0.9	15.3	17.9
SUL	13.7	**13.9**	0.2	13.7	14.0	17.2	**15.9**	0.7	15.1	17.2
HW	5.6	**5.4**	0.2	5.3	5.6	5.8	**5.7**	0.3	5.4	6.1
HDL	5.5	**5.1**	0.4	4.7	5.5	5.6	**5.4**	0.2	5.2	5.6
TD	0.8	**0.8**	0.1	0.7	0.8	1.0	**0.9**	0.1	0.8	1.0
ED	2.0	**1.6**	0.4	1.2	2.0	2.3	**2.1**	0.1	2.0	2.3
IOD	1.4	**1.4**	0.1	1.3	1.4	1.6	**1.6**	0.1	1.4	1.6
EAD	3.0	**2.9**	0.1	2.8	3.0	2.9	**3.0**	0.1	2.8	3.2
EPD	4.6	**4.5**	0.1	4.4	4.6	4.8	**4.7**	0.3	4.3	5.1
IND	2.0	**1.9**	0.1	1.8	2.0	2.2	**2.1**	0.3	1.7	2.5
SL	2.2	**2.1**	0.1	2.1	2.2	2.5	**2.3**	0.2	1.9	2.5
SNL	0.7	**0.7**	0.1	0.7	0.8	1.3	**1.0**	0.2	0.8	1.3
ENL	1.5	**1.4**	0.1	1.3	1.5	1.3	**1.3**	0.2	1.0	1.6
HL	2.8	**2.8**	0.0	2.8	2.8	2.7	**2.8**	0.1	2.7	3.1
RL	3.1	**3.1**	0.0	3.0	3.1	3.6	**3.1**	0.3	2.9	3.6
MD1	0.9	**0.9**	0.1	0.9	1.0	1.3	**1.3**	0.3	1.1	1.9
MD2	1.4	**1.2**	0.1	1.1	1.4	1.4	**1.6**	0.2	1.4	1.9
MD3	1.8	**1.9**	0.1	1.8	2.0	2.5	**2.5**	0.2	2.3	2.8
MD4	1.5	**1.4**	0.1	1.4	1.5	1.5	**1.6**	0.2	1.4	1.9
PD1	0.9	**1.0**	0.2	0.9	1.2	1.0	**1.0**	0.1	0.9	1.2
PD2	1.5	**1.7**	0.2	1.5	1.9	1.6	**1.6**	0.2	1.4	1.9
PD3	3.0	**2.9**	0.0	2.9	3.0	2.7	**3.1**	0.3	2.7	3.5
PD4	4.3	**4.3**	0.0	4.3	4.3	4.7	**4.5**	0.2	4.3	4.7
PD5	2.5	**2.4**	0.1	2.3	2.5	2.8	**2.6**	0.3	2.1	2.9
FL	7.0	**7.2**	0.2	7.0	7.3	7.5	**7.6**	0.2	7.2	7.9
TL	7.0	**7.2**	0.2	7.0	7.4	8.4	**7.7**	0.4	7.2	8.4
FTL	8.4	**7.9**	0.5	7.3	8.4	9.2	**8.5**	0.5	7.9	9.2
IMTL	0.9	**0.9**	0.0	0.9	0.9	0.8	**0.8**	0.1	0.7	1.0
OMTL	0.5	**0.6**	0.1	0.5	0.8	0.5	**0.5**	0.1	0.4	0.6

*Coloration of holotype in life* (Figs. 9A and 9B): Dorsum coloration orange-brown with darker brown eyelids and interorbital region. Dorsal glands outlined by reddish brown. Region under supratympanic fold colored light brown with darker outline of the fold. Under eyes and on the sides are well visible white spots. Venter uniformly light yellow with a couple of symmetrically situated dark and white spots on the chest. Slightly darker gular region bounded by almost white line along the edge of lower jaw. Light yellow color of chest and slightly orange semitransparent ventral skin on front limbs separates sharp boundary. Ventral sides of hind limbs vivid orange with small groups of white spots on the front sides of thighs.

*Coloration of holotype in preservative* (Figs. 9C and 9D): Dorsum light brown with darker eyelids, interorbital region, snout and around dorsal glands. Same darker coloration can be seen under supratympanic fold. Small white spots are visible on the upper lip. Ventral coloration uniformly light yellow with exception of symmetrical dark spots on the chest. Front and hind limbs have same ventral coloration.

*Morphological variation:* The external body-shape morphology is relatively consistent among the individuals; descriptive morphometrics are provided in Table 5. Snout-vent length of males 14.0 and 14.5 mm (*n* = 2) and 15.3–17.9 mm in adult females (*n* = 5). The coloration in life is known only for the female holotype (see above). The coloration variation in preserved specimens resembles the variation of *P. arcanus*. Dorsal glands indistinct. Loreal region lighter than tympanum and canthus rostralis. White spots may be present along the edge of the upper jaw and beneath tympanum. Lightly colored interorbital band may be present. Dark spots placed laterally on each side of the dorsal glands. The wide monochromatic band that extends from the snout tip to the posterior edge of urostyle may be also present. Males with black throat with dark pigmentation extending to the venter and flanks with small white spots. Females with light-yellow colored throat and venter and small white spots on flanks. Some with darker spots in pectoral region (See Fig. 10).

*Cranial osteology:* Male paratype (Figs. 5D–5F) and female holotype (Figs. S3D–S3F) do not differ in general cranial morphology: The ventromedial portion of sphenethmoid fuzed; palatines present but reduced to thin bones only; laterally positioned vomers reduced and lacking teeth-bearing dentigerous processes; maxillary and premaxillary teeth present; alary process of premaxilla inclined laterally; anterior rami of pterygoids separated from maxilla; medial rami of pterygoids reduced in length; no mandibular teeth-like structures present; tip of cultriform process of parasphenoid serrated; body of cultriform process narrowed at its base; parasphenoid alae moderately long and oriented perpendicularly to anteroposterior body axis; only lateral portions of nasals mineralized; nasals not overlapping sphenethmoid, and not in contact medially; otic foramen large; medial anterior margins of frontoparietals extend little bit further than lateral margins; frontoparietals slightly wider posteriorly than anteriorly; ventral ramus of squamosal slightly curved in posterior direction and relatively short; zygomatic rami of squamosal wider and longer than otic rami; anterior processes of quadratojugals laterally overlapped, but not in contact with maxilla; columella relatively gracile.

*Tadpoles:* Are not known.

*Genetics:* The three sequenced specimens included the same haplotype of the 510-bp long 16S rRNA fragment. Average genetic distances are shown in Table 2. Except *P. arcanus*, all other species from the Cameroon radiation differ by 2.8% (*P. chukuchuku*) to 4.9% (*P. manengoubensis*). The sister species, *P. arcanus* differs in 1.2% in the *16S* barcode, and 5.0% in the whole *12S–16S* fragment as a higher number of base-pair differences is present in a hypervariable region of *16S* out of the DNA barcode fragment. The two found haplotypes of the nuclear *RAG1* are unique, different from the haplotypes of *P. arcanus*.

*Advertisement call:* Based on a recording made by J.-L. Amiet on 24 December 1972 as *Phrynobatrachus* sp. 5 (Amiet & Goutte, 2017). Clicking notes of a “wooden” or slightly metallic, tonal and not frequency-modulated sound, relatively fast “tick-tick-tick-tick-tick…tick/tick” (Figs. 7E–7H). Notes are produced in note groups, which are distinguishable by lower amplitude intensity in the beginning and end of a note group, by the last two notes spaced from each other by a shorter time (approx. 70 ms) than are the other notes in the note group (approx. 170 ms), and by the last note with a lower pitch (dominant frequency). Note groups are usually not interspersed by distinct inter-note-group intervals. Note groups (2.2 ± 0.3 s; mean ± standard deviation) consist of 8–11 notes (clicks; 10.3 ± 1.3) with each note 14.8 ± 0.2 ms long. Average note repetition rate is 4.8 ± 0.2 notes per second, repetition rate does not vary substantially during a call (~relatively small standard deviation). The dominant frequency is at 3.51 ± 0.05 kHz.

*Natural history:* The investigated specimens were found in a montane forest near the stream (Fig. 8I) after nightfall (ca. 20:00 to 21:30), active at 20 °C. The female holotype laid a clutch of eggs overnight while in a collecting bottle on 28 January 2000 (middle of dry season). Beside the holotype, which was distinguished based on the egg clutch, the other collected specimens of *P. mbabo* were admixed with juvenile *P. steindachneri* and not directly distinguished in the field as both species live in syntopy and are superficially similar (juvenile *P. steindachneri* and adult *P. mbabo*; see also Fig. S2 for a comparison with *P. arcanus*). Amiet & Goutte (2017) published the advertisement call and described the calling sites as swampy meadows; call activities occurring during daylight, recorded on 24 December 1972 (dry season). It is probable that *P. mbabo* enters montane gallery forests rather rarely, with its typical habitat in open wetlands in montane grasslands, the same what was observed in the sister species, *P. arcanus*.

*Distribution:* Known only from the type locality on Mt. Tchabal Mbabo, Adamawa Region, Cameroon (Figs. 8A, 8G–8I), elevation approx. 2,000 to 2,100 m a.s.l. Tchabal Mbabo is a large, U-shaped massif with relatively steep and forested northern slopes and a main ridge approx. 50 km long. It is possible that *P. mbabo* is distributed in swampy meadows and gallery forests at around 2,000 m a.s.l. along the mountain ridge. A record by Amiet & Goutte (2017) as *Phrynobatrachus* sp. 5 is localized as “Mayo Yim,” which is essentially at same site as the type locality (valley of the Yim stream). This is the only relatively easily accessible area on the ridge of Tchabal Mbabo.

*Threat status:* The species is of a high conservation concern, similar to *P. arcanus* (see above). *Phrynobatrachus mbabo* was not found during a survey in June 2016, thus, the species was last seen in 2000, when the type material was collected. We presume that *P. mbabo* may be underlying a severe population decline like other montane *Phrynobatrachus* in the Cameroon Highlands (Hirschfeld et al., 2016; Doherty-Bone & Gvoždík, 2017; Tchassem et al., 2019). This is probably the reason why it was not found in 2016 as *P. steindachneri* was also not found. Considering its limited range with the known area of occupancy <10 km^2^, a single distribution location, and the presumed population decline (zero specimens found in 2016), we highly recommend to categorize *P. mbabo* as Critically Endangered (following the IUCN criteria; IUCN Standards & Petitions Subcommittee, 2017).

### Dated species tree

**Figure 11:**
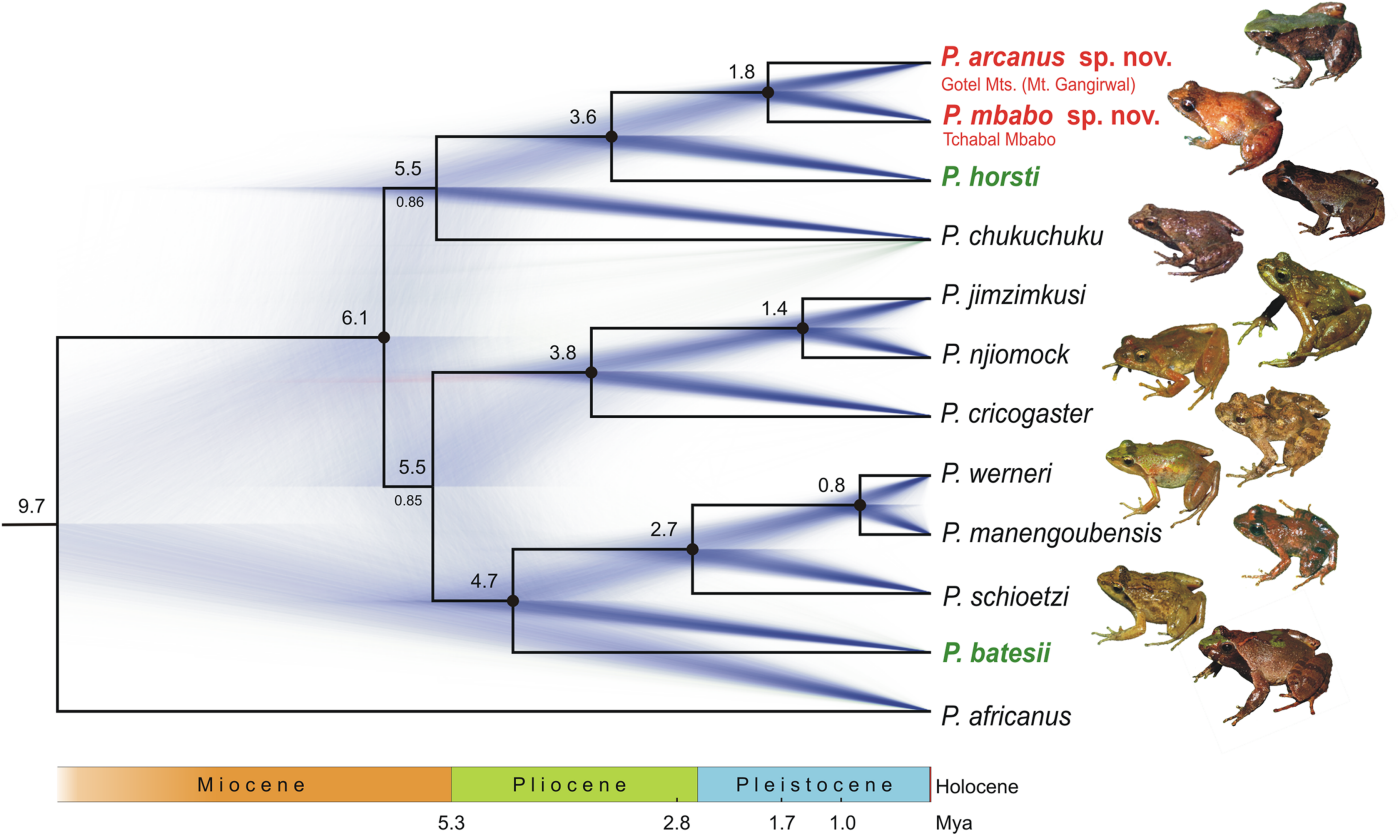
Species tree of the Cameroon radiation of *Phrynobatrachus*. Lowland species in green color, the new lineage/species from the Gotel Mountains and Tchabal Mbabo in red color. The maximum clade credibility tree based on *BEAST analysis of mtDNA and phased nuclear *RAG1* with node ages represented by their median heights (in Mya; numbers above branches) is over-imposed on a ‘cloudogram’ of 27,000 post-burn-in trees. Highly supported nodes denoted by black dots, otherwise posterior probabilities are below branches. Time scale is showing the geological epochs and periods of increased aridity in Africa (DeMenocal, 1995, 2004). Photos by authors, and Marius Burger (*P. horsti*), Thomas M. Doherty-Bone (*P. chukuchuku*), and Daniel M. Portik (*P. schioetzi*, *P. werneri*).

*BEAST analysis inferred a tree (Fig. 11) with the same topology as the mitochondrial tree with the exception of the placement of *P. chukuchuku*, which is inferred with weaker support (0.86) as sister to the highly supported clade (1.00) containing southern lowland *P. horsti* and the two new species from northern mountains. All major clades are highly supported but the relationships among them are not resolved (posterior probabilities 0.85–0.86). The divergence dating analysis puts the origin of the Cameroon radiation to the Miocene–Pliocene boundary 6.1 Mya (median value; 95% HPD: 4.6–7.7 Mya), when the diversifications into major clades occurred. Origins of the species groups were dated to the Pliocene: the *P. werneri* group 4.7 Mya (3.2–6.2 Mya; including *P. batesii*), the *P. steindachneri* group 3.8 Mya (2.3–5.3 Mya; including *P. cricogaster*), and the split between the lowland *P. horsti* and the two northern new species 3.6 Mya (2.3–5.0 Mya). Terminal taxa are of Pleistocene origin, including sister-species pairs: *P. arcanus*–*P. mbabo* 1.8 Mya (0.9–2.8 Mya), *P. jimzimkusi*–*P. njiomock* 1.4 Mya (0.6–2.4 Mya), and *P. werneri*–*P. manengoubensis* 0.8 Mya (0.2–1.4 Mya).

### Species distributions

Genetic data showed that *P. manengoubensis* is more widely distributed than previously known and is also present in the Bakossi Mountains, Cameroon, sharing the same *16S* haplotype as specimens from the type locality, Mt. Manengouba (Zimkus, 2009; Pfalzgraff et al., 2015). For the first time, *P. schioetzi* is confirmed genetically from Cameroon. The specimen from the Bamenda Highlands has a haplotype differing by 0.4% from the haplotype of a specimen from the type locality, Obudu Plateau in Nigeria (Zimkus, 2009; Blackburn & Rödel, 2011). By inclusion of recent data from GenBank, it is demonstrated that all three lowland species are more widespread than previously documented (Fig. S1). *Phrynobatrachus batesii*, *P. horsti*, and *P. ruthbeateae* were confirmed from the Lékoumou Department, Republic of the Congo (Deichmann et al., 2017; specimen numbers: USNM 584173, USNM HerpTissue7, USNM 584174). The three species were not found at the same site, but in the same region (see USNM catalog).

## Discussion

### Phylogeny, taxonomy and biogeography

Phylogenetic analyses of the nuclear *RAG1* gene showed the western Central African *P. africanus*, a species complex that requires additional taxonomic investigation, as the sister lineage to the Cameroon radiation of *Phrynobatrachus*. Both these western Central African/Lower Guinean lineages form a sister clade to some forest West African/Upper Guinean species (Fig. 2). From a biogeographic point of view, this supports a phylogenetic relationship between the Upper and Lower Guinean forests. Phylogenetic analyses of the Cameroon radiation of *Phrynobatrachus* based on mtDNA, nuclear *RAG1* gene, and the Bayesian coalescent-based species tree approach uncovered several highly supported clades, which we propose to treat as species groups. These species groups probably originated during the Miocene–Pliocene transition and subsequently during the Pliocene, when the African climate generally changed to drier conditions and oscillations between arid and humid conditions increased (Ruddiman, 1989). Our dating estimates generally match the dating of split events received for the afrobatrachian frogs (Portik et al., 2019) corresponding to the Miocene–Pliocene radiation of species groups and the Pleistocene age of closely related species pairs.

A new lineage uncovered from the northern mountains of the CVL, named the *P. arcanus* species group, contains two new species from the Gotel Mts. (*P. arcanus*) and Tchabal Mbabo (*P. mbabo*). The two species separated from each other in the Pleistocene ca. 1.8 Mya, which coincides with the increase of aridity in Africa (DeMenocal, 1995; DeMenocal, 2004), and thus, possible disconnections of high-altitude humid habitats, where *P. arcanus* and *P. mbabo* occur. Rather surprisingly, this northernmost montane lineage groups together with the southernmost lowland rainforest lineage, named the *P. ruthbeateae* species group, known from within the northwestern Congolian and Atlantic coastal forests from southern Cameroon and Gabon, to the southern Republic of the Congo (Rödel et al., 2012, 2015; Deichmann et al., 2017; Dewynter & Frétey, 2019). The northern montane and southern lowland species groups separated in the Pliocene (ca. 3.6 Mya).

It is possible that more, yet undescribed species belong to these two species groups. One potential candidate is *Phrynobatrachus* sp. 3 (sensu Amiet, 1978), which morphologically resembles members of the Cameroon radiation, and is known to occur in all savanna zones north of the southern forested region (Amiet & Goutte, 2017). Genetic data is needed from *Phrynobatrachus* specimens from this area of Cameroon to test this hypothesis.

**Figure 12:**
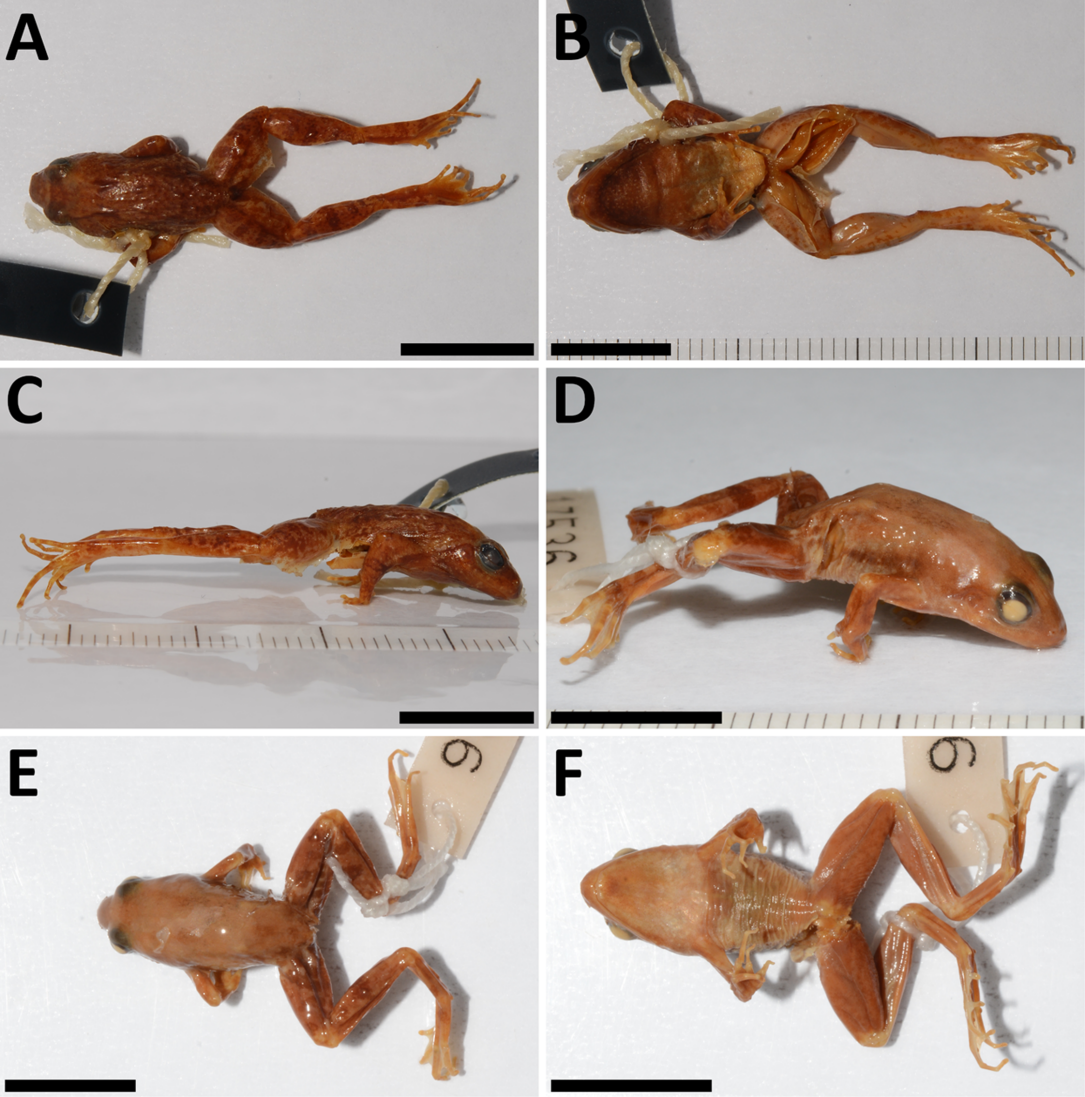
Holotype of *P. hylaios* and syntype of *P. latifrons*. (A–C) Holotype of *P. hylaios* (MHNG 964.100, adult male) in dorsal, ventral and lateral views. (D–F) Syntype of *P. latifrons* (MCZ A-17536, probably adult male) in lateral, dorsal and ventral views. Note the difference in the extent of the pedal webbing. Scale = 10 mm.

*Phrynobatrachus hylaios* is an enigmatic species, which was described from southern Cameroonian rainforests approximately at the border between the Atlantic coastal forests and Congolian forests (Perret, 1959, 1966). DNA sequences of “*P*. cf. *hylaios*” (or “*P*. cf. *hylaois*” sic) deposited in GenBank are of a taxon belonging to the clade containing *P. latifrons* (Zimkus, Rödel & Hillers, 2010), which is morphologically very distinct from the holotype of *P. hylaios*. According to the clearly different morphology, the same is truth for the records of “*P. hylaios*” by Jackson & Blackburn (2007), Jackson et al. (2007; misspelled as “*P. hylaois*”), and Jackson & Blackburn (2010), which are based on misidentifications of *Phrynobatrachus* conforming in general morphology to *P. latifrons*, and in particular to *P. perpalmatus* Boulenger, 1898 (cf. original species description; Boulenger, 1898). One of the clearly distinguishing characters is the extent of the pedal webbing—rudimentary in *P. hylaios* while well developed in *P. latifrons* (Fig. 12) and *P. perpalmatus* (Boulenger, 1898). Pending a thorough revision, and if we consider the phylogenetic diversity of the clade containing *P. latifrons* (Zimkus, Rödel & Hillers, 2010) and the type locality of *P. perpalmatus* (Lake Mweru, Zambia–Democratic Republic of the Congo/DRC), we tentatively propose to re-identify “*P*. aff. *latifrons*” (from DRC and Tanzania) and “*P*. cf. *hylaios*” (from Republic of the Congo and Central African Republic) sensu Zimkus, Rödel & Hillers (2010), Zimkus et al. (2012) and Deichmann et al. (2017) as *P. perpalmatus* and *P*. cf. *perpalmatus*, respectively. Similarly, “*P. hylaios*” sensu Jackson & Blackburn (2007), Jackson et al. (2007) and Jackson & Blackburn (2010) corresponds to *P*. cf. *perpalmatus*, a putative new species probably closely related to *P. perpalmatus*.

The holotype of *P. hylaios* is morphologically most similar to *P. horsti* (Perret, 1959, 1966; Rödel et al., 2015). Perret (1966) hypothesized that *P. hylaios* is distributed from southern Cameroon to Bas-Congo (western DRC) and possibly to Uélé (northern DRC). The species has been recorded in the southwestern Republic of the Congo (Largen & Dowsett-Lemaire, 1991), a region from where *P. horsti* was later described (Rödel et al., 2015). The latter was recorded also from Gabon (Haut–Ogooué Province), however, initially as *P. ruthbeateae* (Zimkus & Gvoždík, 2013; Zimkus & Larson, 2013a; 2013b). An additional record from Haut-Ogooué, Gabon, was published also by Jongsma et al. (2017). A photo of a male of “*P. ruthbeateae*” from central Gabon published by Dewynter & Frétey (2019) is most probably either *P. horsti* or *P. hylaios*. Given the similarity of general morphology and color pattern between *P. hylaios* and *P. horsti*, the type specimens must be investigated thoroughly to understand the level of distinction of the two taxa.

**Figure 13:**
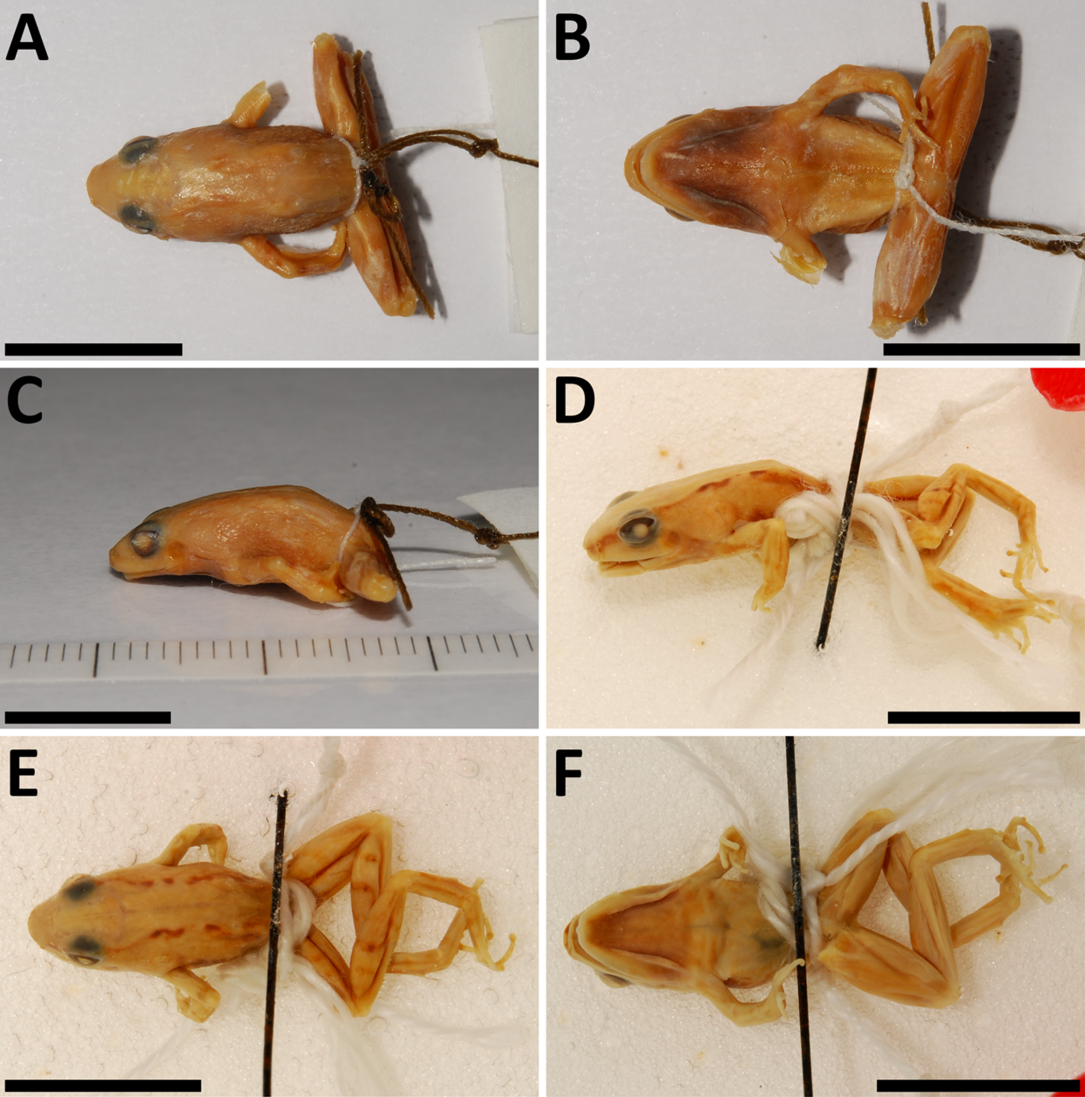
Lectotype of *P. werneri* and holotype of *P. manengoubensis*. (A–C) Lectotype of *P. werneri* (ZMB 20434, adult male) in dorsal, ventral and lateral views. (D–F) Holotype of *P. manengoubensis* (MNHN 1939-113 (1599-1), adult male) in lateral, dorsal and ventral views. Note the general similarity of the two taxa and similar structure of the vocal sac (distinct folds along mandibles). Scale = 10 mm.

*Phrynobatrachus hylaios* was initially described as a subspecies of *P. werneri* (Perret, 1959). Amiet & Goutte (2017) also proposed that *P. hylaios* is morphologically and bioacoustically close to *P. werneri* and that a subspecific status might be more appropriate. We disagree with this statement for a number of reasons: (1) *P. hylaios* represents a lowland rainforest species, while *P. werneri* is a (sub)montane species, (2) the general morphologies and color patterns of the types are different (Figs. 12A–12C and 13A–13C), and (3) the structure of vocal sac of males is very different (Figs. 12B and 13B; Perret, 1966). It is unlikely that “*P. hylaios*” from Tibati (savanna zone in Adamawa, Cameroon) pictured by Amiet & Goutte (2017) represents *P. hylaios*, rather this likely should be identified as *P. werneri*. Tibati is approx. 100 km from the type locality of *P. werneri* in the vicinity of Banyo. However, the presented recordings of the advertisement calls of *P. hylaios* originated from the southern forested region, thus, probably conform to this species (Amiet & Goutte, 2017). For the time being, and based on the above evidence, we consider *P. hylaios* a member of the *P. ruthbeateae* species group, probably closely related to *P. horsti*. Furthermore, we propose that the only locality record without doubt is the type locality (Foulassi, Sangmelima, alt. 710 m a.s.l., Cameroon; Perret, 1959), and probably also Ototomo and Zamakoé from the south of Yaoundé (Amiet & Goutte, 2017). The identification of “*P. hylaios*” from Mt. Nlonako by Herrmann et al. (2005) also remains questionable based on the biogeographic patterns (mountains north of the Sanaga River harbor usually different evolutionary lineages in amphibians; Portik et al., 2017; Charles et al., 2018; Leaché et al., 2019) until the specimen is thoroughly investigated. Last, “*P. hylaios*” on the photograph in Channing & Rödel (2019) also does not correspond to the morphology of *P. hylaios* (e.g., missing dark face mask).

*Phrynobatrachus chukuchuku*—a species with uncertain phylogenetic affinity—probably originated during the Miocene–Pliocene transition, ca. 5.5 Mya. It is known only from high elevations of Mt. Oku (Zimkus, 2009; Pfalzgraff et al., 2015; Doherty-Bone & Gvoždík, 2017). On Mt. Oku, there is another, yet undescribed species present, *Phrynobatrachus* sp. aff. *werneri* (Doherty-Bone & Gvoždík, 2017), which is probably conspecific with *Phrynobatrachus* sp. 4 (sensu Amiet, 1978) known also from Mt. Bamboutos and Mt. Lefo/Santa (Amiet & Goutte, 2017). It inhabits wet meadows, and occasionally forests (Doherty-Bone & Gvoždík, 2017) at the elevation range 1,800 to 2,800 m a.s.l. (Amiet & Goutte, 2017; Doherty-Bone & Gvoždík, 2017). Its phylogenetic position is not yet known but it is possible that is allied to either *P. chukuchuku* or the *P. werneri* species group.

The *P. werneri* species group, originated probably during the Miocene–Pliocene boundary (ca. 4.7 Mya), is easily recognizable in *16S* by a unique insertion (4–7 bp), which is present in *P. batesii* but absent in *P. chukuchuku* (sometimes clustering together with the *P. werneri* group in the sister position). Therefore, we consider *P. batesii* a member of the *P. werneri* group but not *P. chukuchuku*.

There has been a long debate on the systematic position of *P. manengoubensis*, if it is conspecific with or distinct from *P. werneri* (Grandison, 1985; Zimkus, 2009; Blackburn, 2010; Amiet & Goutte, 2017). There are no substantial morphological differences except of the body size (*P. manengoubensis* being smaller; Fig. 13), however, there is a genetic divergence (relatively shallow, split dated to 0.8 Mya) between the two taxa and unique haplotypes in *RAG1*. For the time being, we continue to treat *P. manengoubensis* as a distinct, though relatively young, species probably originating during the last major Pleistocene aridification in Africa (DeMenocal, 1995; DeMenocal, 2004). Based on mtDNA there is a support for a wider distribution of *P. manengoubensis* that includes the Bakossi Mountains in addition to the type locality on Mt. Manengouba. A phylogeographic approach to study the relationship between *P. werneri* and *P. manengoubensis* must be applied to understand the evolution of these two closely related taxa.

The remaining species within the *P. werneri* species group include *P. schioetzi*, *P. danko*, and *P. batesii. Phrynobatrachus schioetzi* is genetically confirmed from Cameroon (Bamenda Highlands) for the first time, although it was previously reported from this locality without genetic confirmation (Doherty-Bone & Gvoždík, 2017). *Phrynobatrachus danko* is still known only from the type locality on the Mambilla Plateau in Nigeria. The lowland forest species, *P. batesii*, which is sister to all remaining taxa in the *P. werneri* group, is known from southeastern Nigeria to Gabon. Records from West Africa are probably based on misidentifications according to differences in the advertisement call as previously discussed (Amiet & Goutte, 2017). Dewynter & Frétey (2019) suggested that although the species probably occurs in northern Gabon, all records previously assigned to Gabon were based on misidentifications. However, they overlooked the finding of Deichmann et al. (2017) that the species was recently confirmed even from the southern Republic of the Congo, Lékoumou Department, from near the Gabonese border (ca. 40 km). This indicates that *P. batesii* is likely present throughout Gabon.

We include *P. cricogaster*, a largely submontane species (Gartshore, 1986), within the *P. steindachneri* species group as this species is recovered in the sister position to the remaining taxa from the group (split dated to the Pliocene, ca. 3.8 Mya). We recognize all the predominantly montane taxa and evolutionary lineages that were previously named as “*P. steindachneri*” within this species group: *P. jimzimkusi*, *P. njiomock*, and *P. steindachneri* A, B, C, D. All these taxa/evolutionary lineages are of relatively young age and originated during the Pleistocene climatic oscillations. This species complex is subjected to a separate study (M. Dolinay & V. Gvoždík, 2019, unpublished data).

### Advertisement calls

If we evaluate the known advertisement calls of *Phrynobatrachus* as presented in a monograph on advertisement calls of Cameroonian frogs by Amiet & Goutte (2017), the most similar calls to our two new species can be found in *P. hylaios*, *P. werneri*, and *P. manengoubensis*. Similarities can be found also in *P. horsti* from the western Congo (Rödel et al., 2015; recordings of “*P. hylaios*” by Largen & Dowsett-Lemaire (1991) are most probably conspecific to the later described *P. horsti*). Advertisement calls of *P. arcanus*, *P. mbabo*, and the above-listed species can be described as tonal and not frequency-modulated clicking notes sounding like “tick tick tick…” emitted at different paces depending on the species. Another common characteristics of these species is probably predominantly diurnal calling activity (see Amiet & Goutte, 2017). All these species are members of the Cameroon radiation, which fits expectations that phylogenetically closely related anurans tend to have similar types of advertisement calls (Gingras et al., 2013). The *P. ruthbeateae* species group (containing *P. horsti* and most probably also *P. hylaios*; the species with the most similar calls) was uncovered in our analyses as the sister clade to *P. arcanus* and *P. mbabo*. The more distantly related *P. werneri* and *P. manengoubensis* have advertisement calls still similar but more different (see Amiet & Goutte, 2017). However, to understand the evolution of advertisement calls, one would need acoustic data collected from genotyped specimens of all species across a clade.

### Conservation remarks

Both new species, *P. arcanus* and *P. mbabo*, are known only from the type localities from high elevations in montane grasslands with a mosaic of stream-side fringing gallery forests, but no specimens were found during recent field surveys of the type localities in 2016. *Phrynobatrachus arcanus* was seen in 2009 and *P. mbabo* in 2000 for the last time. This may indicate that the new species are underlying population declines, similarly to other montane *Phrynobatrachus* species from the CVL (Hirschfeld et al., 2016; Doherty-Bone & Gvoždík, 2017; Tchassem et al., 2019). The known declines in *Phrynobatrachus* are still evaluated as “enigmatic”, and researchers are not sure what is happening with montane anurans in the Cameroon Highlands. The declines, at least in some regions, are probably a result of a combination of more negative factors like land use by humans, including forest loss and degradation, usage of agricultural chemicals or expanding aquaculture (Doherty-Bone & Gvoždík, 2017; Tchassem et al., 2019). These factors can be important in more densely populated places, such as Mt. Oku or Mt. Bamboutos. However, the remote places with a low human population density, like the Gotel Mts. or Tchabal Mbabo, do not fit to these presumptions. Except for livestock grazing, which has been the traditional way of life in the region for centuries, it seems that an increased agricultural encroachment is not an issue in the Gotel Mts. and Tchabal Mbabo. However, from preliminary results, it seems that the main reason of the amphibian declines in the remote northern mountains is a spread of the amphibian chytrid pathogen (V. Gvoždík & V. Baláž, 2019, unpublished data), which is consistent also with findings made in more southern mountains, mainly Mt. Manengouba (Hirschfeld et al., 2016). In any case, these negative findings deserve more attention and further highlight the urgent necessity of conservation interventions in the mountains of Cameroon and Nigeria.

## Conclusions

The phylogeny, taxonomy, and biogeography of the Cameroon radiation of puddle frogs (*Phrynobatrachus*) was studied, identifying a novel evolutionary lineage from the northern part of the CVL based on the morphologic and molecular investigations. Two new species were described within the new lineage, *P. arcanus* sp. nov. from the Gotel Mountains (Cameroon–Nigeria border), and *P. mbabo* sp. nov. from Tchabal Mbabo (Cameroon). Interestingly, the new lineage is phylogenetically close to the recently described taxa from lowland rainforests (*P. horsti*, *P. ruthbeateae*). We proposed five species groups within the Cameroon radiation (*P. arcanus*, *P. chukuchuku*, *P. ruthbeateae*, *P. steindachneri*, and *P. werneri*) with the basal diversification event dated to the late Miocene, subsequent diversifications to the Pliocene, and closely related terminal taxa, including the new species, are dated to originate during the Pleistocene climatic oscillations. We discuss the possible position of *P. hylaios* within the *P. ruthbeateae* species group given that no molecular data is currently available. A phylogeographic approach should be applied also to understand the evolutionary relationship between *P. werneri* and *P. manengoubensis*, two closely related taxa, with the latter shown to be more widely distributed across the CVL (not confined only to the type locality as previously assumed). We note that the *P. steindachneri* species complex requires further attention. In addition, we provided new observations to confirm that *Phrynobatrachus* is disappearing from the Cameroon Highlands, with an important agent responsible for the population declines could be attributed to the amphibian chytrid pathogen (V. Gvoždík & V. Baláž, 2019, unpublished data). Therefore, and due to the limited distribution ranges, the two new species are recommended to be evaluated as Critically Endangered. This further highlights the urgent necessity of conservation research and actions in the montane ecosystems of the CVL.

## Supplemental Information

10.7717/peerj.8393/supp-1 Supplemental Information 1 Acustic recording.

10.7717/peerj.8393/supp-2 Supplemental Information 2 Supplemental Information

10.7717/peerj.8393/supp-3 Supplemental Information 3 Table S1. Measurements (in mm) of *P. arcanus* sp. nov. For measurement abbreviations see “Materials and Methods”, male holotype and female allotype in bold.

10.7717/peerj.8393/supp-4 Supplemental Information 4 Table S2. Measurements (in mm) of *P. mbabo* sp. nov. For measurement abbreviations see “Materials and Methods”, male allotype and female holotype in bold.

10.7717/peerj.8393/supp-5 Supplemental Information 5 New sequences.

## Additional Information and Declarations

### Field Study Permissions

The following information was supplied relating to field study approvals (i.e., approving body and any reference numbers): Research and collecting permits were issued by the Cameroon Ministry of Scientific Research and Innovation (MINRESI: No. 00039/MINRESI/B00/C00/C10/C12) and the Ministry of Forests and Wildlife (MINFOF: Nos. 1010/PRBS/MINFOF/SG/DFAP/SDVEF/SC and 1099/PRS/MINFOF/SG/DFAP/SDVEF/SC).

### Data Availability

The following information was supplied regarding data availability: The molecular data is available at GenBank: MN158662–MN158695. The morphometric data is available in Tables S1 and S2. The acoustic recording is available both as a Supplemental File and at FonoZoo: 11489 (only available by request from the database). The raw μCT data is available at MorphoSource: 27195, 27196, 27198 and 27200. https://www.morphosource.org/Detail/SpecimenDetail/Show/specimen_id/27195 https://www.morphosource.org/Detail/SpecimenDetail/Show/specimen_id/27196 https://www.morphosource.org/Detail/SpecimenDetail/Show/specimen_id/27198 https://www.morphosource.org/Detail/SpecimenDetail/Show/specimen_id/27200 The specimens are stored at the National Museum in Prague (NMP); Zoologisches Forschungsmuseum Alexander Koenig, Bonn (ZFMK); Museum für Naturkunde Berlin (ZMB); and the Institut National de Recherche en Sciences Exactes et Naturelles, Brazzaville (IRSEN): *P. arcanus* sp. nov. NMP-P6V 74603/1; *P. arcanus* sp. nov. NMP-P6V 74603/2; *P. arcanus* sp. nov. NMP-P6V 74603/3; *P. arcanus* sp. nov. NMP-P6V 74603/4; *P. arcanus* sp. nov. NMP-P6V 74603/5; *P. arcanus* sp. nov. NMP-P6V 74603/6; *P. arcanus* sp. nov. NMP-P6V 74603/7; *P. arcanus* sp. nov. NMP-P6V 74603/8; *P. arcanus* sp. nov. NMP-P6V 74604; *P. arcanus* sp. nov. NMP-P6V 75866/A; *P. arcanus* sp. nov. NMP-P6V 75866/B; *P. arcanus* sp. nov. NMP-P6V 75866/C; *P. arcanus* sp. nov. ZFMK 47955; *P. arcanus* sp. nov. ZFMK 47956; *P. arcanus* sp. nov. ZFMK 47957; *P. arcanus* sp. nov. ZFMK 47958; *P. mbabo* sp. nov. ZFMK 75726; *P. mbabo* sp. nov. ZFMK 75676; *P. mbabo* sp. nov. ZFMK 75686; *P. mbabo* sp. nov. ZFMK 75650; *P. mbabo* sp. nov. ZFMK 75677; *P. mbabo* sp. nov. ZFMK 75683; *P. mbabo* sp. nov. ZFMK 75728; *P. mbabo* sp. nov. ZFMK 75729; *P. batesii* NMP-P6V 74620; *P. cricogaster* NMP-P6V 73393/1; *P. cricogaster* NMP-P6V 73393/5; *P. cricogaster* NMP-P6V 74642; *P. horsti* ZMB 81699; *P. horsti* IRSEN-MBUR 3028; *P. horsti* IRSEN-MBUR 3029; *P. horsti* IRSEN-MBUR 3030; *P. horsti* IRSEN-MBUR 3031; *P. manengoubensis* NMP-P6V 74607; *P. manengoubensis* NMP-P6V 74644; *P. schioetzi* NMP-P6V 73438.

### New Species Registration

The following information was supplied regarding the registration of a newly described species: Publication LSID: urn:lsid:zoobank.org:pub:71CF018D-9E8B-46CD-A6EA-1F9CCB809765. Phrynobatrachus arcanus sp. nov. LSID: urn:lsid:zoobank.org:act:C9921DC5-52CE-4CDF-A0A5-AE77D044299A. Phrynobatrachus mbabo sp. nov. LSID: urn:lsid:zoobank.org:act:422C3BB8-B16E-48ED-97FB-88D9142FEEDC.

## References

[ref-1] Altig R (2007). A primer for the morphology of anuran tadpoles. Herpetological Conservation and Biology.

[ref-2] Altig R, McDiarmid RW, McDiarmid RW, Altig R (1999). Body plan: development and morphology. Tadpoles: The Biology of Anuran Larvae.

[ref-3] Amiet J-L (1975). Ecologie et distribution des Amphibiens Anoures de la région de Nkongsamba (Cameroun). Annales de la Faculte des Sciences de Yaoundé.

[ref-4] Amiet J-L (1978). Liste provisoire des amphibiens anoures du Cameroun.

[ref-5] Amiet J-L (2004). Phrynobatrachus manengoubensis. http://dx.doi.org/10.2305/IUCN.UK.2004.RLTS.T58125A11736272.en.

[ref-6] Amiet J-L, Goutte S (2017). Chants d’Amphibiens du Cameroun.

[ref-7] Angel F (1940). Description de trois amphibiens nouveaux du Cameroun, matériaux de la mission P. Lepesme, R. Paulian et A. Villiers (2^e^ note). Bulletin du Muséum national d’histoire naturelle, série.

[ref-8] Blackburn DC (2010). A new puddle frog (Phrynobatrachidae: *Phrynobatrachus*) from the Mambilla Plateau in Eastern Nigeria. African Journal of Herpetology.

[ref-9] Blackburn DC, Rödel M-O (2011). A new puddle frog (Phrynobatrachidae: *Phrynobatrachus*) from the Obudu Plateau in eastern Nigeria. Herpetologica.

[ref-10] Bouckaert RR, Heled J (2014). DensiTree 2: seeing trees through the forest. bioRxiv 012401.

[ref-11] Boulenger GA (1898). Fourth report on additions to the batrachian collection in the Natural-History Museum.

[ref-12] Burgess N, Hales JDA, Underwood E, Dinerstein E, Olson D, Itoua I, Schipper J, Ricketts T, Newman K (2004). Terrestrial ecoregions of Africa and Madagascar: a conservation assessment.

[ref-13] Burke K (2001). Origin of the Cameroon Line of volcano-capped swells. Journal of Geology.

[ref-14] Böhme W, Nikolaus G (1989). Herpetological specimens from the Gotel Mountains and Mambilla Plateau. Nigeria Tauraco Research Report.

[ref-15] Castresana J (2000). Selection of conserved blocks from multiple alignments for their use in phylogenetic analysis. Molecular Biology and Evolution.

[ref-86] Center for Conservation Bioacoustics (2016). http://ravensoundsoftware.com.

[ref-87] Channing A, Rödel M-O (2019). Field guide to the frogs & other amphibians of Africa.

[ref-16] Channing A, Rödel M-O, Channing J (2012). Tadpoles of Africa: the biology and identification of all known tadpoles in sub-Saharan Africa.

[ref-17] Charles KL, Bell RC, Blackburn DC, Burger M, Fujita MK, Gvoždík V, Jongsma GFM, Kouete MT, Leaché AD, Portik DM (2018). Sky, sea, and forest islands: diversification in the African leaf-folding frog *Afrixalus paradorsalis* (Anura: Hyperoliidae) of the Lower Guineo-Congolian rain forest. Journal of Biogeography.

[ref-18] Cronin DT, Libalah MB, Bergl RA, Hearn GW (2014). Biodiversity and conservation of tropical montane ecosystems in the Gulf of Guinea, West Africa. Arctic, Antarctic, and Alpine Research.

[ref-19] Darriba D, Taboada GL, Doallo R, Posada D (2012). jModelTest 2: more models, new heuristics and parallel computing. Nature Methods.

[ref-20] Deichmann JL, Mulcahy DG, Vanthomme H, Tobi E, Wynn AH, Zimkus BM, McDiarmid RW (2017). How many species and under what names? Using DNA barcoding and GenBank data for west Central African amphibian conservation. PLOS ONE.

[ref-21] DeMenocal PB (1995). Plio-Pleistocene African climate. Science.

[ref-22] DeMenocal PB (2004). African climate change and faunal evolution during the Pliocene–Pleistocene. Earth and Planetary Science Letters.

[ref-23] Dewynter M, Frétey T (2019). Liste taxonomique commentée et catalogue illustré des Amphibiens du Gabon. Les Cahiers de la Fondation Biotope.

[ref-24] Doherty-Bone TM, Gvoždík V (2017). The Amphibians of Mount Oku, Cameroon: an updated species inventory and conservation review. ZooKeys.

[ref-25] Drewes RC (1984). A phylogenetic analysis of the Hyperoliidae (Anura): treefrogs of Africa, Madagascar, and the Seychelles Islands. Occasional Papers of the California Academy of Sciences.

[ref-26] Drummond AJ, Bouckaert RR (2015). Bayesian evolutionary analysis with BEAST.

[ref-27] Drummond AJ, Ho SYW, Phillips MJ, Rambaut A (2006). Relaxed phylogenetics and dating with confidence. PLOS Biology.

[ref-28] Drummond AJ, Suchard MA, Xie D, Rambaut A (2012). Bayesian phylogenetics with BEAUti and the BEAST 1.7. Molecular Biology and Evolution.

[ref-29] Duellman WE, Trueb L (1986). Biology of Amphibians.

[ref-30] Frost DR (2019). http://research.amnh.org/herpetology/amphibia/index.html.

[ref-31] Gartshore ME, Stuart SN (1986). The status of the montane herpetofauna of the Cameroon Highlands. Conservation of Cameroon Montane Forests.

[ref-32] Gingras B, Mohandesan E, Boko D, Fitch WT (2013). Phylogenetic signal in the acoustic parameters of the advertisement calls of four clades of anurans. BMC Evolutionary Biology.

[ref-33] Gosner KL (1960). A simplified table for staging anuran embryos and larvae with notes on identification. Herpetologica.

[ref-34] Grandison AGC, Frost DR (1985). Phrynobatrachus. Amphibian Species of the World: A Taxonomic and Geographical Reference.

[ref-35] Gridi-Papp M (2007). SoundRuler: acoustic analysis for research and teaching. http://www.soundruler.sourceforge.net.

[ref-36] Guindon S, Dufayard J-F, Lefort V, Anisimova M, Hordijk W, Gascuel O (2010). New algorithms and methods to estimate maximum-likelihood phylogenies: assessing the performance of PhyML 3.0. Systematic Biology.

[ref-37] Heled J, Drummond AJ (2010). Bayesian inference of species trees from multilocus data. Molecular Biology and Evolution.

[ref-38] Herrmann H-W, Böhme W, Herrmann PA, Plath M, Schmitz A, Solbach M (2005). African biodiversity hotspots: the amphibians of Mt. Nlonako, Cameroon. Salamandra.

[ref-39] Herrmann H-W, Schmitz A, Herrmann PA, Böhme W (2007). Amphibians and reptiles of the Tchabal Mbabo Mountains, Adamaoua Plateau, Cameroon. Bonner Zoologische Beiträge.

[ref-40] Hirschfeld M, Blackburn DC, Doherty-Bone TM, Gonwouo LN, Ghose S, Rödel M-O (2016). Dramatic declines of montane frogs in a Central African biodiversity hotspot. PLOS ONE.

[ref-41] Huelsenbeck JP, Rannala R (2004). Frequentist properties of Bayesian posterior probabilities of phylogenetic trees under simple and complex substitution models. Systematic Biology.

[ref-42] IUCN Standards and Petitions Subcommittee (2017). http://www.iucnredlist.org/documents/RedListGuidelines.pdf.

[ref-43] Jackson K, Blackburn DC (2007). The amphibians and reptiles of Nouabale-Ndoki National Park, Republic of Congo (Brazzaville). Salamandra.

[ref-44] Jackson K, Blackburn DC (2010). A survey of amphibians and reptiles at degraded sites near Pointe-Noire, Kouilou Province, Republic of Congo. Herpetological Conservation and Biology.

[ref-45] Jackson K, Zassi-Boulou A-G, Mavoungou L-B, Pangou S (2007). Amphibians and reptiles of the Lac Télé Community Reserve, Likouala Region, Republic of Congo (Brazzaville). Herpetological Conservation and Biology.

[ref-46] Jongsma GFM, Tobi E, Dixon-MacCallum GP, Bamba-Kaya A, Yoga J-A, Mbega J-D, Mve Beh J-H, Emrich AM, Blackburn DC (2017). Amphibians of Haut-Ogooué Province, southeastern Gabon. Amphibian & Reptile Conservation.

[ref-47] Katoh K, Standley DM (2013). MAFFT multiple sequence alignment software version 7: improvements in performance and usability. Molecular Biology and Evolution.

[ref-48] Kocher TD, Thomas WK, Meyer A, Edwards SV, Pääbo S, Villablanca FX, Wilson AC (1989). Dynamics of mitochondrial DNA evolution in animals: amplification and sequencing with conserved primers. Proceedings of the National Academy of Sciences of the United States of America.

[ref-49] Kumar S, Stecher G, Li M, Knyaz C, Tamura K (2018). MEGA X: molecular evolutionary genetics analysis across computing platforms. Molecular Biology and Evolution.

[ref-50] Köhler J, Jansen M, Rodríguez A, Kok PJR, Toledo LF, Emmrich M, Glaw F, Haddad CFB, Rödel M-O, Vences M (2017). The use of bioacoustics in anuran taxonomy: theory, terminology, methods and recommendations for best practice. Zootaxa.

[ref-51] Lanfear R, Frandsen PB, Wright AM, Senfeld T, Calcott B (2017). PartitionFinder 2: new methods for selecting partitioned models of evolution for molecular and morphological phylogenetic analyses. Molecular Biology and Evolution.

[ref-52] Largen MJ, Dowsett-Lemaire F (1991). Amphibians (Anura) from the Kouilou River basin, République du Congo. Tauraco Research Report.

[ref-53] Leaché AD, Portik DM, Rivera D, Rödel M-O, Penner J, Gvoždík V, Greenbaum E, Jongsma GFM, Ofori-Boateng C, Burger M, Eniang EA, Bell RC, Fujita MK (2019). Exploring rain forest diversification using demographic model testing in the African foam-nest treefrog *Chiromantis rufescens*. Journal of Biogeography.

[ref-54] Lefort V, Longueville JE, Gascuel O (2017). SMS: smart model selection in PhyML. Molecular Biology and Evolution.

[ref-55] Librado P, Rozas J (2009). DnaSP v5: a software for comprehensive analysis of DNA polymorphism data. Bioinformatics.

[ref-56] Marzoli A, Piccirillo EM, Renne P, Bellieni G, Iacumin M, Nyobe NB, Tongwa AT (2000). The Cameroon Volcanic Line revisited: petrogenesis of continental basaltic magmas from lithospheric and asthenospheric mantle sources. Journal of Petrology.

[ref-57] Mbowou GIB, Lagmet C, Nomade S, Ngounouno I, Deruelle B, Ohnenstetter D (2012). Petrology of the Late Cretaceous peralkaline rhyolites (pantellerite and comendite) from Lake Chad, Central Africa. Journal of GEOsciences.

[ref-58] Metscher BD (2009). MicroCT for developmental biology: a versatile tool for high-contrast 3D imaging at histological resolutions. Developmental Dynamics.

[ref-59] Nieden F (1910). Neue Reptilien und Amphibien aus Kamerun. Archiv für Naturgeschichte.

[ref-60] Palumbi S, Martin A, Romano S, McMillan WO, Stice L, Grabowski G (1991). The simple fool’s guide to PCR.

[ref-61] Perret J-L (1959). Études herpétologiques africains. Bulletin de la Société Neuchâteloise des Sciences Naturelles.

[ref-62] Perret J-L (1966). Les Amphibiens du Cameroun. Zoologische Jahrbücher. Abteilung für Systematik, Ökologie und Geographie.

[ref-63] Pfalzgraff T, Hirschfeld M, Barej MF, Dahmen M, Gonwouo LN, Doherty-Bone TM (2015). The tadpoles of four Central Africa *Phrynobatrachus* species. Salamandra.

[ref-64] Portik DM, Bell RC, Blackburn DC, Bauer AM, Barratt CD, Branch WR, Burger M, Channing A, Colston TJ, Conradie W, Dehling JM, Drewes RC, Ernst R, Greenbaum E, Gvoždík V, Harvey J, Hillers A, Hirschfeld M, Jongsma GFM, Kielgast J, Kouete MT, Lawson LP, Leaché AD, Loader SP, Lötters S, Van Der Meijden A, Menegon M, Müller S, Nagy ZT, Ofori-Boateng C, Ohler A, Papenfuss TJ, Rößler D, Sinsch U, Rödel M-O, Veith M, Vindum J, Zassi-Boulou A-G, McGuire JA (2019). Sexual dichromatism drives diversification within a major radiation of African amphibians. Systematic Biology.

[ref-65] Portik DM, Leaché AD, Rivera D, Barej MF, Burger M, Hirschfeld M, Rödel M-O, Blackburn DC, Fujita MK (2017). Evaluating mechanisms of diversification in a Guineo-Congolian tropical forest frog using demographic model selection. Molecular Ecology.

[ref-66] Rambaut A, Drummond AJ, Xie D, Baele G, Suchard MA (2018). Posterior summarization in Bayesian phylogenetics using Tracer 1.7. Systematic Biology.

[ref-67] Richards CM, Moore WS (1996). A phylogeny for the African treefrog family Hyperoliidae based on mitochondrial rDNA. Molecular Phylogenetics and Evolution.

[ref-68] Ronquist F, Teslenko M, Van Der Mark P, Ayres DL, Darling A, Höhna S, Larget B, Liu L, Suchard MA, Huelsenbeck JP (2012). MrBayes 3.2: efficient Bayesian phylogenetic inference and model choice across a large model space. Systematic Biology.

[ref-69] Ruddiman WF (1989). Late Miocene to Pleistocene evolution of climate in Africa and the low-latitude Atlantic: overview of Leg 108 results. Proceedings of the Ocean Drilling Program: Scientific Results.

[ref-70] Rödel M-O, Burger M, Zassi-Boulou AG, Emmrich M, Penner J, Barej M (2015). Two new *Phrynobatrachus* species (Amphibia: Anura: Phrynobatrachidae) from the Republic of the Congo. Zootaxa.

[ref-71] Rödel M-O, Doherty-Bone T, Kouete MT, Janzen P, Garrett K, Browne R, Gonwouo NL, Barej MF, Sandberger L (2012). A new small *Phrynobatrachus* (Amphibia: Anura: Phrynobatrachidae) from southern Cameroon. Zootaxa.

[ref-72] Sabaj MH (2019). https://asih.org/standard-symbolic-codes/about-symbolic-codes.

[ref-73] Stephens M, Scheet P (2005). Accounting for decay of linkage disequilibrium in haplotype inference and missing-data imputation. American Journal of Human Genetics.

[ref-74] Stephens M, Smith JN, Donnelly P (2001). A new statistical method for haplotype reconstruction from population data. American Journal of Human Genetics.

[ref-75] Stuart SN (1986). Conservation of Cameroon Montane Forests.

[ref-76] Tchassem FAM, Doherty-Bone TM, Kameni NMM, Tapondjou NWP, Tamesse JL, Gonwouo LN (2019). What is driving declines of montane endemic amphibians? New insights from Mount Bamboutos, Cameroon. Oryx.

[ref-77] Vences M, Nagy ZT, Sonet G, Verheyen E, Kress WJ, Erickson DL (2012). DNA barcoding amphibians and reptiles, DNA Barcodes: methods and protocols. Methods in Molecular Biology.

[ref-78] White F (1983). The vegetation of Africa: a descriptive memoir to accompany the UNESCO/AETFAT/UNSO vegetation map of Africa.

[ref-79] Zimkus BM (2009). Biogeographical analysis of Cameroonian puddle frogs and description of a new species of *Phrynobatrachus* (Anura: Phrynobatrachidae) endemic to Mount Oku. Cameroon Zoological Journal of the Linnean Society.

[ref-80] Zimkus BM, Gvoždík V (2013). Sky Islands of the Cameroon Volcanic Line: a diversification hot spot for puddle frogs (Phrynobatrachidae: *Phrynobatrachus*). Zoologica Scripta.

[ref-81] Zimkus BM, Larson JG (2013a). *Phrynobatrachus ruthbeatae* [sic] (Ruth-Beate’s Puddle Frog). Gabon: Haut Ogooé Province: Batéké Plateau National Park. Herpetological Review.

[ref-82] Zimkus BM, Larson JG (2013b). Assessment of the amphibians of Batéké Plateau National Park, Gabon, including results of chytrid pathogen tests. Salamandra.

[ref-83] Zimkus BM, Lawson LP, Barej MF, Barratt CD, Channing A, Dash KM, Dehling JM, Du Preez L, Gehring P-S, Greenbaum E, Gvoždík V, Harvey J, Kielgast J, Kusumba C, Nagy ZT, Pabijan M, Penner J, Rödel M-O, Vences M, Letters S (2017). Leapfrogging into new territory: how Mascarene ridged frogs diversified across Africa and Madagascar to maintain their ecological niche. Molecular Phylogenetics and Evolution.

[ref-84] Zimkus BM, Lawson L, Loader SP, Hanken J (2012). Terrestrialization, miniaturization and rates of diversification in African puddle frogs (Anura: Phrynobatrachidae). PLOS ONE.

[ref-85] Zimkus BM, Rödel M-O, Hillers A (2010). Complex patterns of continental speciation: molecular phylogenetics and biogeography of sub-Saharan puddle frogs (*Phrynobatrachus*). Molecular Phylogenetics and Evolution.

