# Supplementary material for: Evolutionary history of the Cameroon radiation of puddle frogs (Phrynobatrachidae: Phrynobatrachus), with descriptions of two critically endangered new species from the northern Cameroon Volcanic Line
Source: PeerJ. 2020 Mar 3;8:e8393. doi: 10.7717/peerj.8393 (PMC7059761; doi:10.7717/peerj.8393)
Supplement: Supplemental Information 2 [file peerj-08-8393-s002.pdf]

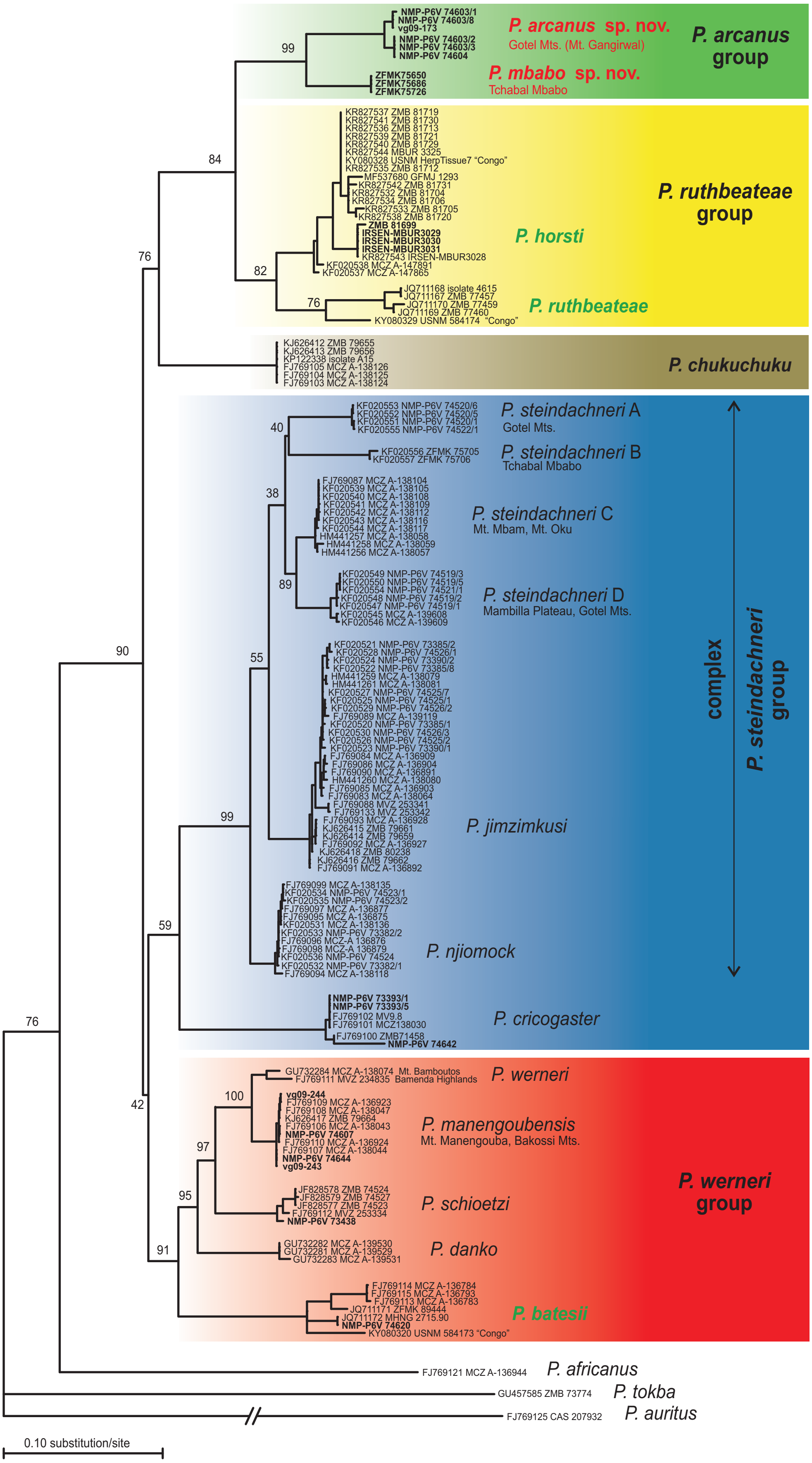

Figure S1. Maximum likelihood tree based on the *individuals-mt-dataset* (*12S–16S*) of the Cameroon radiation of *Phrynobatrachus*. Numbers near nodes are ML bootstrap support values. Background color denotes species groups. Names of lowland species in green color, and the new lineage/species from the Gotel Mountains and Tchabal Mbabo in red color. Specimen codes not starting with NMP, vg, ZFMK, IRSEN, or ZMB are GenBank accession numbers followed by specimen voucher numbers. Specimen codes in bold are new material. Geographic origin of closely related *P. werneri* and *P. manengoubensis*, and *P. steindachneri* is indicated, as of the three lowland species (green) found in the southern Republic of the Congo (Deichmann et al., 2017) marked as “Congo”.

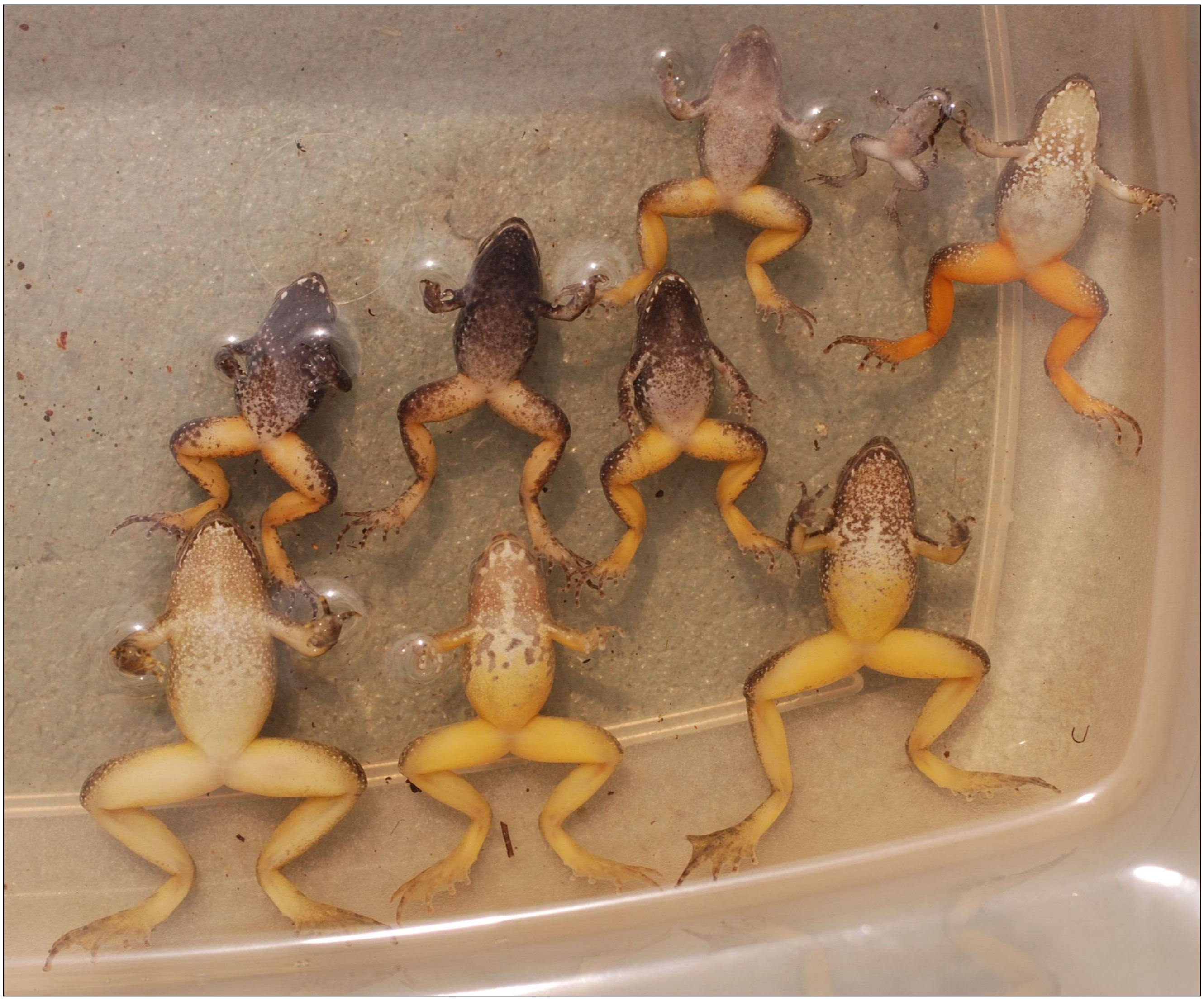

Figure S2. ***Phrynobatrachus arcanus*** **sp. nov. in comparison with subadult** ***P. steindachneri*****.** Both species from the same site – the type locality of *P. arcanus* sp. nov. Note the extended pedal webbing in *P. steindachneri* – the three bottom specimens (from left to right: NMP-P6V 74520/3, 74520/2, 74520/4); while the remaining specimens are *P. arcanus* sp. nov. with only rudimental pedal webbing (the only adult female in the top right position).

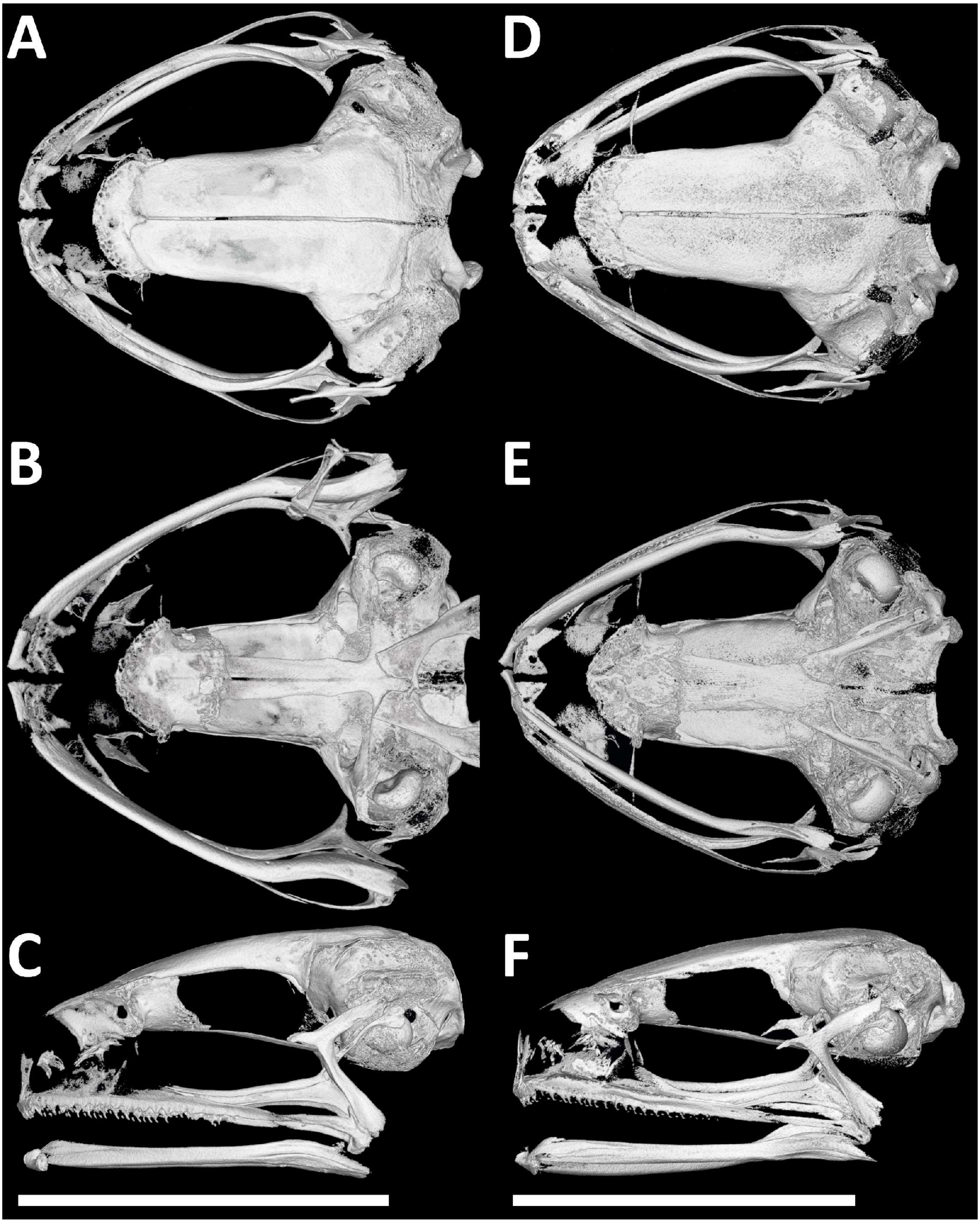

Figure S3. Cranial morphology (µCT scans) of females in dorsal, ventral and lateral views. (A–C) *P. arcanus* sp. nov. (allotype), and (D–F) *P. mbabo* sp. nov. (holotype). Scale = 5 mm.

Table S1. Measurements (in mm) of *P. arcanus* sp. nov.

For measurement abbreviations see Materials & Methods, male holotype and female allotype in bold.

Museum	NMP-P6V						ZFMK	NMP-P6V	ZFMK			NMP-P6V	
Acc. No.	**74603/1**	74603/3	74603/4	74603/5	74603/6	74604	47958	**74603/2**	47955	47956	47957	74603/7	74603/8
Sex/Stage	**male**	male	male	male	male	male	male	**female**	female	female	female	juvenile	juvenile
SVL	**13.8**	17.0	14.6	14.6	14.1	14.5	13.1	**14.8**	16.5	15.8	16.9	7.4	8.7
SUL	**13.6**	16.3	14.3	14.5	13.4	14.1	13.0	**14.3**	15.9	15.7	16.5	-	-
HW	**5.2**	5.6	5.4	5.4	5.6	5.3	5.0	**5.8**	5.5	5.5	5.5	-	-
HDL	**4.9**	5.3	5.2	5.3	4.8	4.9	4.9	**5.1**	5.7	5.5	5.3	-	-
TD	**1.1**	0.8	1.0	0.9	0.9	1.0	0.9	**1.0**	0.9	0.8	1.1	-	-
ED	**1.8**	2.2	1.9	2.0	1.9	1.9	2.1	**2.0**	1.9	2.2	2.2	-	-
IOD	**1.2**	1.9	1.7	1.7	1.8	1.6	1.8	**1.5**	1.7	1.8	1.8	-	-
EAD	**2.7**	3.0	2.9	2.8	3.0	2.5	2.6	**2.9**	2.9	2.8	2.8	-	-
EPD	**4.5**	4.4	4.7	4.4	4.5	4.2	4.1	**4.1**	4.9	5.0	4.8	-	-
IND	**2.3**	2.2	2.4	2.2	2.2	2.1	1.9	**2.0**	2.1	2.2	2.1	-	-
SL	**1.9**	2.5	1.9	1.9	1.8	2.0	2.2	**2.5**	2.2	1.9	2.2	-	-
SNL	**0.6**	0.8	1.0	0.6	0.6	0.8	1.1	**1.0**	1.0	1.0	1.3	-	-
ENL	**1.4**	1.7	1.0	1.2	1.2	1.2	1.0	**1.5**	1.2	0.9	0.9	-	-
HL	**2.8**	3.0	2.1	2.3	2.1	2.1	2.4	**2.4**	3.0	2.8	3.0	-	-
RL	**3.4**	2.9	3.1	3.0	3.0	2.6	2.6	**3.1**	2.9	2.7	2.7	-	-
MD1	**1.5**	1.1	1.0	0.8	1.0	1.0	0.9	**1.0**	1.4	1.3	1.6	-	-
MD2	**1.5**	1.1	1.1	1.3	1.2	1.1	1.1	**1.2**	1.4	1.4	1.7	-	-
MD3	**2.5**	2.2	1.9	1.8	1.3	2.2	1.7	**1.9**	2.5	2.3	2.4	-	-
MD4	**1.2**	1.4	1.2	1.4	1.0	1.3	1.1	**1.0**	1.3	1.3	1.4	-	-
PD1	**1.3**	0.9	0.7	0.8	0.5	0.7	0.6	**0.8**	1.0	0.7	1.2	-	-
PD2	**1.8**	1.8	1.2	1.1	1.0	1.0	1.1	**1.2**	1.6	1.4	1.9	-	-
PD3	**2.6**	2.9	2.5	2.1	2.1	2.4	2.2	**2.3**	3.4	3.0	2.5	-	-
PD4	**4.5**	4.7	3.9	4.2	4.1	3.7	3.9	**4.3**	5.0	4.6	4.8	-	-
PD5	**2.1**	2.2	2.0	2.1	2.0	2.0	2.1	**2.1**	2.9	2.3	2.4	-	-
FL	**6.6**	7.6	6.6	7.1	6.7	6.7	6.0	**7.2**	7.2	6.5	7.5	-	-
TL	**7.1**	7.3	6.9	7.6	7.1	7.0	6.3	**7.3**	6.1	6.2	7.3	-	-
FTL	**8.8**	7.8	7.0	7.5	6.8	6.7	6.6	**7.0**	6.8	7.2	7.4	-	-
IMTL	**0.8**	1.0	1.0	1.1	1.0	0.8	0.8	**0.8**	0.7	0.7	0.8	-	-
OMTL	**0.7**	0.6	0.4	0.6	0.5	0.4	0.3	**0.5**	0.4	0.4	0.5	-	-

Table S2. Measurements (in mm) of *P. mbabo* sp. nov.

For measurement abbreviations see Materials & Methods, male allotype and female holotype in bold.

Museum				ZFMK				
Acc. No.	**75676**	75683	**75726**	75650	75677	75686	75728	75729
Sex/Stage	**male**	male	**female**	female	female	female	female	subadult
SVL	**14.0**	14.5	**17.9**	16.2	15.8	15.3	16.3	10.9
SUL	**13.7**	14.0	**17.2**	15.8	15.6	15.1	15.7	-
HW	**5.6**	5.3	**5.8**	5.5	6.1	5.5	5.4	-
HDL	**5.5**	4.7	**5.6**	5.2	5.4	5.2	5.6	-
TD	**0.8**	0.7	**1.0**	0.9	1.0	0.8	0.9	-
ED	**2.0**	1.2	**2.3**	2.0	2.1	2.3	2.0	-
IOD	**1.4**	1.3	**1.6**	1.6	1.4	1.5	1.6	-
EAD	**3.0**	2.8	**2.9**	3.2	2.9	3.1	2.8	-
EPD	**4.6**	4.4	**4.8**	4.3	5.1	4.8	4.5	-
IND	**2.0**	1.8	**2.2**	1.7	2.5	2.0	2.4	-
SL	**2.2**	2.1	**2.5**	1.9	2.5	2.3	2.4	-
SNL	**0.7**	0.8	**1.3**	0.9	0.9	1.0	0.8	-
ENL	**1.5**	1.3	**1.3**	1.0	1.6	1.3	1.6	-
HL	**2.8**	2.8	**2.7**	3.1	2.7	2.7	2.9	-
RL	**3.1**	3.0	**3.6**	3.0	3.0	2.9	3.0	-
MD1	**0.9**	1.0	**1.3**	1.2	1.1	1.3	1.9	-
MD2	**1.4**	1.1	**1.4**	1.4	1.9	1.5	1.7	-
MD3	**1.8**	2.0	**2.5**	2.3	2.5	2.5	2.8	-
MD4	**1.5**	1.4	**1.5**	1.4	1.6	1.8	1.9	-
PD1	**0.9**	1.2	**1.0**	0.9	0.9	1.2	1.1	-
PD2	**1.5**	1.9	**1.6**	1.4	1.9	1.8	1.7	-
PD3	**3.0**	2.9	**2.7**	2.9	3.0	3.5	3.2	-
PD4	**4.3**	4.3	**4.7**	4.3	4.3	4.7	4.7	-
PD5	**2.5**	2.3	**2.8**	2.1	2.4	2.9	2.8	-
FL	**7.0**	7.3	**7.5**	7.2	7.7	7.5	7.9	-
TL	**7.0**	7.4	**8.4**	7.2	7.7	7.7	7.7	-
FTL	**8.4**	7.3	**9.2**	8.6	7.9	7.9	8.7	-
IMTL	**0.9**	0.9	**0.8**	0.8	1.0	0.8	0.7	-
OMTL	**0.5**	0.8	**0.5**	0.5	0.6	0.5	0.4	-
